# S100A4 Is a Key Facilitator of Thoracic Aortic Dissection

**DOI:** 10.7150/ijbs.83091

**Published:** 2024-01-01

**Authors:** Jiajun Shi, Wenjun Yu, Chuan Liang, Hongjie Shi, Dengwei Cao, Yong Ran, Haisen Qiao, Zhe Dong, Jinping Liu

**Affiliations:** 1 Department of Cardiovascular Surgery, Zhongnan Hospital of Wuhan University, Wuhan 430071, China.; 2 Hubei Provincial Engineering Research Center of Minimally Invasive Cardiovascular Surgery, Wuhan 430071, China.; 3 Wuhan Clinical Research Center for Minimally Invasive Treatment of Structural Heart Disease, Wuhan 430071, China.

**Keywords:** S100A4, Thoracic aortic dissection, Lysyl oxidase, Vascular smooth muscle cells, Extracellular matrix

## Abstract

**Background:** Thoracic aortic dissection (TAD) is one of the cardiovascular diseases with high incidence and fatality rates. Vascular smooth muscle cells (VSMCs) play a vital role in TAD formation. Recent studies have shown that extracellular S100A4 may participate in VSMCs regulation. However, the mechanism(s) underlying this association remains elusive. Consequently, this study investigated the role of S100A4 in VSMCs regulation and TAD formation.

**Methods:** Hub genes were screened based on the transcriptome data of aortic dissection in the Gene Expression Synthesis database. Three-week-old male S100A4 overexpression (AAV9- S100A4 OE) and S100A4 knockdown (AAV9- S100A4 KD) mice were exposed to β-aminopropionitrile monofumarate through drinking water for 28 days to create the murine TAD model.

**Results:** S100A4 was observed to be the hub gene in aortic dissection. Furthermore, overexpression of S100A4 was exacerbated, whereas inhibition of S100A4 significantly improved TAD progression. In the TAD model, the S100A4 was observed to aggravate the phenotypic transition of VSMCs. Additionally, lysyl oxidase (LOX) was an important target of S100A4 in TAD. S100A4 interacted with LOX in VSMCs, reduced mature LOX (m-LOX), and decreased elastic fiber deposition, thereby disrupting extracellular matrix homeostasis and promoting TAD development. Elastic fiber deposition in human aortic tissues was negatively correlated with the expression of S100A4, which in turn, was negatively correlated with LOX.

**Conclusions:** Our data showed that S100A4 modulates TADprogression, induces lysosomal degradation of m-LOX, and reduces the deposition of elastic fibers by interacting with LOX, thus contributing to the disruption of extracellular matrix homeostasis in TAD. These findings suggest that S100A4 may be a new target for the prevention and treatment of TAD.

## Introduction

Thoracic aortic dissection (TAD), the tearing of the intima of the blood vessels, is caused by the separation of the aortic wall and has a high fatality rate 1. When surgery is inapplicable, TAD progresses to rupture with high mortality 2. Despite the long-awaited non-surgical treatment of TAD, few options are available because the molecular pathogenesis of TAD is unclear. Therefore, the etiology and molecular pathogenesis of TAD must be studied to provide new treatment options to prevent or delay TAD progression.

TAD is characterized by the transformation of vascular smooth muscle cells (VSMCs) phenotype and disruption of extracellular matrix (ECM) homeostasis, causing aortic aneurysms, dissection, and rupture 3. VSMCs in the aorta have a complex array of phenotypes, including contractile phenotypes that regulate blood flow and pressure and the synthetic phenotype of the dedifferentiated state 4. VSMCs with contractile phenotypes delay TAD progression 5,6 and other cardiovascular diseases 7,8. In addition, elastic fibers are the largest structure and the most stable ECM component that confers essential resiliency to the aortic vessel wall 9,10. Identifying the specific mechanisms of VSMC phenotypic transition and ECM homeostasis disruption is necessary to develop new TAD prevention and treatment approaches.

We screened the hub genes associated with aortic dissection using the Gene Expression Omnibus (GEO) database to identify the target genes associated with TAD pathogenesis. S100A4 is encoded by the S100A4 gene in humans located within a frequently rearranged gene cluster on chromosome 1q21 11 and is expressed in cancer cells and vascular cells, among others 12. S100A4 is significantly elevated in tumor cells 13, and it activates collagenase 3 transcriptional activity 14, thereby enhancing tumor invasion and metastasis 13. Moreover, during the transformation of endothelial cells into mesenchymal, S100A4 is expressed as a mesenchymal cell-specific gene and is protein encoding 15. In addition, recent studies have shown that extracellular S100A4 can induce tumor cells to produce pro-inflammatory cytokines 16,17 and converts VSMCs to a pro-inflammatory phenotype, which contributes to the formation and rupture of atherosclerotic plaques 18. The differential expression of S100A4 has been reported in both TAD 19 and abdominal aortic aneurysm 20. Moreover, S100A4 expression is up-regulated in TAD 21. However, the effect of S100A4 on TAD formation and the specific mechanism requires further investigation.

In this study, we utilized AAV-S100A4 overexpressing and AAV-S100A4 knockdown mice to examine the impact of S100A4 on the formation and progression of TAD. Furthermore, employing a multidisciplinary translational approach, we aimed to validate the hypothesis that targeting S100A4 could be a potential therapeutic strategy for attenuating TAD progression.

## Methods

### Human aorta samples and ethics statement

All protocols using human specimens were approved by the Ethics Committee of Zhongnan Hospital Wuhan University, Hubei, China and performed in compliance with the principles of the Declaration of Helsinki. All participating patients provided informed signed consent. TAD samples were obtained from patients with thoracic aortic repair or replacement, and control samples were obtained from age-matched organ donors with no history of aortic disease and surgery. Aortic tissue was stored within 2 hours following collection. Briefly, the aorta was cleaned with normal saline to remove fat and blood clots. Once dissected, the tissue was formalin-fixed for 24 hours or frozen in liquid nitrogen and stored at -80°C for subsequent experiments.

### Animal experiments

All animal experiments were performed following the regulations approved by the Ethics Committee of the Animal Experiment Center of Wuhan University under specific pathogen-free barrier conditions.

The TAD mouse model was established with the administration of β-aminopropionitrile monofumarate (BAPN; Sigma-Aldrich, St Louis). C57BL/6J male mice at 21 days of age were exposed to water containing 0.1% or 0.25% BAPN for 4 weeks, and the water was changed daily. The age of the mouse was a key factor in the modeling of BAPN, which promotes elastic fiber degradation leading to TAD formation only when the mouse is in the early stages of rapid growth 22.

Adeno-associated virus subtype 9, including AAV9-GFP, A overexpression (AAV9-A OE), AAV9-Ctrl, and A knockout (AAV9-A KO), were purchased from Wako (Shandong, China). The viruses (8.0×10^11^ VG/ml, 200 ul/mouse) were delivered via tail veins when the mice were 18 days old, and the animals were euthanized 4 weeks later. The aorta was dissected from the ascending aorta to the iliac artery, and excess blood inside and around the vessel was washed gently using a cold PBS solution administered with a syringe. In the process of isolating the media layer of blood vessels, we used the microscope to peel off the endothelium and outer membrane, to obtain a purer media layer. The tissues were placed on the operating sheet and photographed with a scanner. The aorta tissues were stored at -80°C for subsequent western blot (WB) and real-time polymerase chain reaction (RT-PCR) analyses. The maximum diameter of the thoracic aorta was measured using ImageJ as described in previous studies 23. Aortic dissection was defined as hematoma within the aortic wall upon macroscopic examination or delamination (with a false lumen hematoma) in the aortic media upon histological examination.

### Cell isolation, culture, and transfection

Mouse primary VSMCs were harvested from 8-weeks-old mice. Mouse thoracic aorta tissues were removed, the endothelium and adventitia were also gently removed, and the tissue was cut into uniformly sized (1‒2 mm) explants and digested with a combination of 0.2% collagenase type II (Sigma, C6885) and 0.1% type II elastase (Worthington Biochem, LS002292) at 37°C for 1 hour. VSMCs were cultured in high-glucose Dulbecco's Modified Eagle Medium (DMEM) (Gibco, C11995500BT) with 15% fetal bovine serum (Gibco, A3160802), with the medium being changed twice a week. According to previous studies 24, VSMCs were incubated with 250 ug/ml BAPN in DMEM and harvested after 24 hours of stimulation for further analysis. Cells between passages one and three were used in this study.

Mouse S100A4 and LOX cDNA were purchased from Shanghai GeneChem Co., Ltd. Mouse cDNA was cloned into CV702 by Shanghai GeneChem Co., Ltd. Small interfering RNA (siRNA) was designed against S100A4 by Ribobio (Guangzhou, China). In addition, VSMCs were transfected with S100A4 plasmids or S100A4 siRNA for 24 hours via Lipofectamine 3000 (Invitrogen).

### Hematoxylin and eosin (H&E) and elastic fiber staining

Formalin was used to fix the aortic tissue for 24 hours. Subsequently, the aortic tissues were dehydrated with alcohol according to the concentration gradient, embedded in paraffin for sectioning, and sliced with a microtome for 5 μm. Subsequently, the sections were stained with H&E, and Elastic Van Gieson (EVG). All images were captured using a scanner (3DHISTECH CaseViewer; Hungary). The following criteria to determine the level of elastin degradation 25,26: grade 1, no degradation; grade 2, mild degradation; grade 3, severe degradation; and grade 4, aortic rupture.

### Immunofluorescence (IF) staining and imaging

The aortic tissue was fixed in 4% paraformaldehyde solution for 24 hours, shielded from light, and subsequently embedded in paraffin. Slices were cut and immersed in xylene thrice, lasting 15 min each time. Subsequently, they were hydrated with a gradient alcohol solution. The sodium citrate antigen repair buffer (pH=6.0) was prepared, and the tissue slides were immersed in it at 94°C for 20 min. After cooling to room temperature, slides were washed thrice with 1x PBS (+0.2% Triton-X100), lasting 5 min each time. Regarding cellular IF, VSMCs were fixed in 4% paraformaldehyde solution at room temperature for 15 min and permeabilized within 1x PBS (+ 0.2% Triton-X100). The slices were incubated at room temperature for 30 min in 3% BSA in 1x PBS to block non-specific binding. Subsequently, slices were incubated overnight at 4°C with the primary antibodies. The next day, after washing with PBS, luciferin-conjugated secondary antibodies were used to incubate slices at room temperature for 3 hours. After washing thrice, DAPI staining was performed, followed by another round of PBS washing thrice for 5 min each. Furthermore, slides were mounted within an antifade medium. The observation was performed using a fluorescence microscope (3DHISTECH CaseViewer, Hungary) or confocal microscope (SP8, Leica, Germany). Finally, ImageJ software was used for image analysis, and the number of cells and total nuclei in each image was quantified using DAPI staining.

### Protein extraction, immunoprecipitation (IP), and WB

The tissue or resuspended cells were lysed with cold RIPA buffer (Beyotime) and freshly prepared protease inhibitors (Thermo Scientific, 78334). The total lysate was loaded onto SDS-PAGE and transferred to PVDF membranes (Millipore). The membranes were blocked with 5% skim milk and 0.5% Tween20, and then incubated with primary antibody at 4°C overnight. Primary antibodies for WB are listed in Table S3. Subsequently, the membranes were incubated with the corresponding secondary antibodies for 2 hours at room temperature. The bands were visualized using the Enhanced Chemiluminescence system (Tanon 4600). ImageJ software was used to quantify the intensity of the blots.

### RNA isolation and RT-PCR

Total mRNA was isolated using TRIzol per the manufacturer's instructions (Invitrogen Life, 15596-026), was checked for quality and quantified using NanoDrop One (Thermo Fisher Scientific, Madison, WI, USA). Total mRNA was converted to cDNA using HiScript III RT SuperMix (R323-01; Vazyme, Nanjing, China). Following reverse transcription, subsequent experiments were conducted using ChamQ SYBR Master Mix (Vazyme, Q311-02) and Real-Time PCR (Bio-Rad). GAPDH was used as the internal reference. Gene expression was quantitatively analyzed using the 2^-ΔΔCT^ method. All primers used in RT-PCR are shown in Table S5.

### IP assays

VSMCs were transfected with labeled plasmids for 24 hours, then lysed in 500 ul of Tris-HCl, NaCl, ethylenediaminetetraacetic acid, Triton X-100, and freshly prepared protease inhibitors (Thermo Scientific, 78334) for co-IP assays. Aortic tissues were lysed with cold IP buffer and broken using a tissue grinder at 60 Hz for 300 s. Then the sample was centrifuged at 12,000 × g for 10 min. The supernatants were incubated with 20 µL protein A+G magnetic beads (P2108, Beyotime, Shanghai, China) and 1 µg corresponding antibody at 4°C overnight. Subsequently, the beads were washed, then boiled with 2x SDS loading buffer, and the samples were stored at -20°C for subsequent WB analysis. The immunoprecipitated complexes were separated by SDS-PAGE and set up at Shanghai Applied Protein Technology Co., Ltd., Shanghai, China for IP mass spectrometry analysis.

### LOX relative activity assay

A semi-quantitative activity assay was performed using a LOX Activity Assay Kit (ab112139, Abcam). First, we collected the VSMCs culture supernatant and centrifuged the samples. After centrifugation, the supernatant was diluted with PBS+0.1% bovine serum albumin to 50 ul. Subsequently, we mixed 20 ul 250X HRP substrate stock solution and 5 ml assay buffer to prepare the assay reaction mix. The fluorescence at 530-570/590-600 nm was measured after incubation at 37°C for 30 min.

### Serum S100A4 analysis

The blood was left at room temperature for 20 min, then centrifuged at 3,000 rpm for 10 min at 4°C. The serum was transferred to a cryopreservation tube and stored at -80°C. S100A4 in human serum (HM10944, Bio-Swamp, Wuhan, China) and S100A4 in mouse (MU32968, Bio-Swamp, Wuhan, China) serum were determined using a commercial kit. The content of S100A4 in serum was detected using sandwich ELISA according to the kit instructions. First, the standard sample was diluted, and 50 ul was added to the standard well. Subsequently, 40 ul of the sample was added to the sample well and 10 ul of the labeled antibody. Additionally, 50 ul of enzyme-conjugate reagent was added and incubated at 37°C for 30 min. Absorbance was measured at 450 nm wavelength after washing and color rendering.

### Statistical analysis

The results were expressed as mean ± SEM and analyzed using GraphPad Prism (version 8.0). A comparison of means between the two groups was performed using an unpaired Student's t-test. One-way analysis of variance (ANOVA) or the Kruskal-Wallis test was used to perform comparisons between multiple groups. When the variances were equal, ANOVA followed by Tukey's post-test was used for pairwise comparisons. When the variances were unequal, the non-parametric Kruskal-Wallis test and Dunn's multiple comparison procedure were used. A probability value of P<0.05 was considered significant.

## Results

### The expression of S100A4 was elevated in patients and mice with TAD

**Figure 1:**
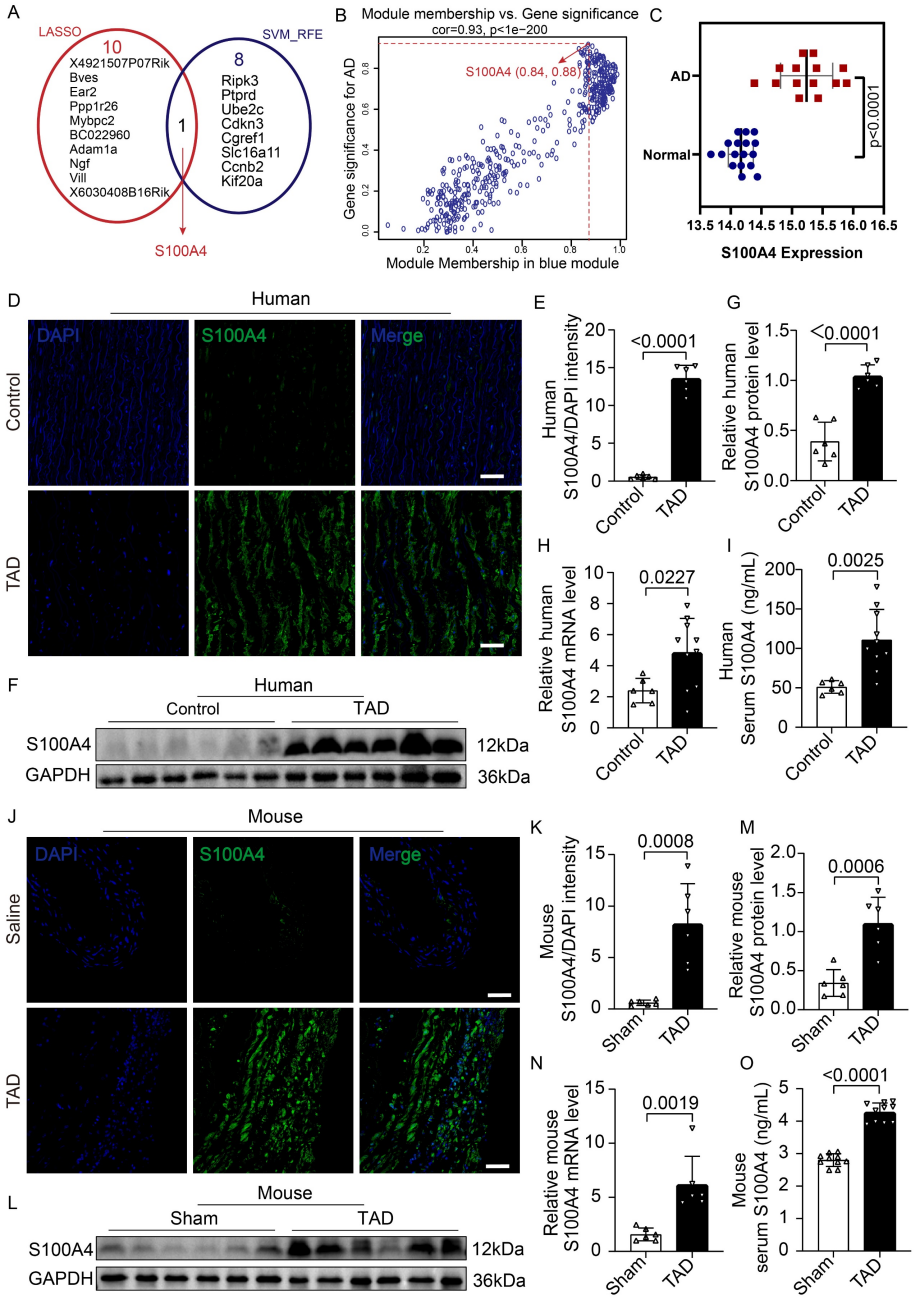
** The expression of S100A4 is increased in mice and patients with TAD. A,** LASSO and SVM-RFE algorithms were used to select the key genes within gene modules correlated with AD (turquoise and blue modules). **B,** Scatter plot of gene significance for AD (aortic dissection) and the module membership in the blue module. **C,** Difference of S100A4 expression between AD and normal tissue (p<0.0001).** D-E,** Immunofluorescence staining to confirm the level of S100A4 in human aortas from controls and patients with TAD (n=6). Scale bars: 50 μm. **F-G,** Immunoblot analysis and quantification of S100A4 levels in aortic wall from controls and patients with TAD (n=6). **H,** RT-PCR analysis of S100A4 in the aortic wall from controls (n=6) and patients with TAD (n=10). **I,** Determination of serum S100A4 content in controls (n=6) and patients with TAD (n=10). **J-K,** Immunofluorescence staining to confirm the level of S100A4 in aortas from healthy control and TAD mice (n=6). Scale bars: 50 μm. **L-M,** Immunoblot analysis and quantification of S100A4 levels in the aortic wall from control and TAD mice (n=6). **N,** RT-PCR analysis of S100A4 in the aortic wall from control and TAD mice (n=6). **O,** Determination of serum S100A4 content in control and TAD mice (n=10).

To screen potential pathogenic genes of TAD, the “WGCNA” algorithm was used to identify key gene modules that were highly correlated with aortic dissection (Figure S1A-S1E). Genes from turquoise (Figure S1D) and blue modules (Figure S1D) were selected for LASSO and SVM-RFE analysis due to their higher correlation with aortic dissection (turquoise module: R = -0.9, p = 2e - 11; blue module: R = 0.75, p = 2e - 06) (Figure S1D). Furthermore, 11 and 9 hub genes were identified according to LASSO and SVM-RFE analysis, respectively (Figure 1A). Hub genes refer to the key pathogenic genes identified by screening. Interestingly, S100A4 was the only gene in the intersection between biomarkers derived from these two algorithms and showed a higher module membership rank in the blue module (module membership = 0.84, gene significance = 0.88) (Figure 1B). Further analysis demonstrated that S100A4 was significantly overexpressed in mice with aortic dissection (Figure 1C). Gene ontology analysis showed that the S100A4 gene was related to ECM, blood vessel morphogenesis, the vascular process in the circulatory system, and smooth muscle cell migration (Figure S2A-S2B).

To further explore the relationship between S100A4 and TAD, we compared the expression of S100A4 between aorta samples of control and patients with TAD. IF staining (Figure 1D, 1E), WB analysis (Figure 1F, 1G), RT-PCR assays (Figure 1H), and ELISA testing (Figure 1I) consistently demonstrated that the expression of S100A4 was low or undetectable in the control aortic tissue, while the expression of S100A4 was significantly increased in the aortic tissue of patients with TAD, which was consistent with the results of previous studies 27-29. Similarly, the expression of S100A4 was significantly up-regulated in TAD mice treated with 0.25% BAPN (Figure 1J-1O). This finding further verified the results of our bioinformatics analysis that S100A4 was significantly overexpressed in TAD.

### S100A4 exacerbates the development of BAPN-induced TAD formation

**Figure 2:**
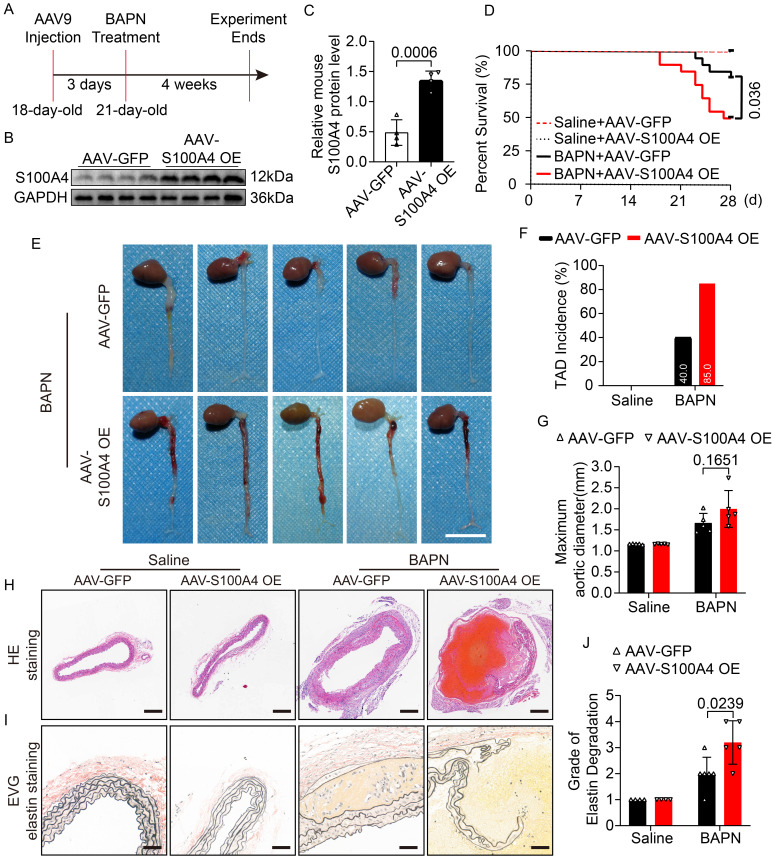
** The overexpression of S100A4 aggravates TAD formation and rupture.** Mice were injected with AAV9-GFP or AAV9-S100A4 OE intravenously at 18 days old and given 0.1%BAPN or equivalent saline for 4 weeks starting at 21 days of age (n=20 per group). **A,** Schematic protocol. **B-C,** Immunoblot analysis and quantification of S100A4 protein in aortic tissue from AAV9-GFP and AAV9-S100A4 OE mice (n=4). **D,** Survival curves in indicated groups (n=20 per group), log-rank test. **E,** Representative images of aortas. Scale bar: 1 cm. **F,** TAD incidence (n=20 per group). **G,** Measurements of maximum aortic diameter (n=4-6, normalized to body weight). **H,** Representative macroscopic images of ascending aorta sections stained with hematoxylin and eosin (H&E). Scale bars: 200 μm. **I,** Representative images of ascending aorta sections stained with Elastic-Van Gieson (EVG). Scale bars: 50 μm. **J,** Grade of elastin degradation in the aortic wall (n=4-6).

Mice were injected with AAV9-GFP and AAV9-S100A4 OE via the caudal vein at 18 days of age to investigate the effect of S100A4 on TAD formation. Previous studies have shown that 0.25% BAPN used *in vivo* has a high incidence of TAD and a high mortality rate 6, which are not conducive to clearly showing the effect of S100A4 overexpression on TAD. Therefore, either 0.1% BAPN or an equal amount of normal saline was added to their drinking water at 21 days of age (Figure 2A). The BAPN and normal saline were changed daily for the following 4 weeks 6,23. We first verified that AAV9-S100A4 OE was efficiently infected and subsequently increased the expression of S100A4 in mouse arterial tissues (Figure 2B, 2C; Figure S3A). Subsequently, at 21 days of age, AAV-GFP and AAV-S100A4 OE mice were given 0.1% BAPN or equal amounts of normal saline in water for 4 weeks. It should be noted that the drinking water of mice containing BAPN or saline needs to be freshly prepared daily. In mice given normal saline, overexpression of S100A4 did not affect the maximum diameter of the aorta, the degradation of elastic fibers, or the incidence of TAD. However, overexpression of S100A4 exacerbated aortic dilatation, TAD incidence, and mortality in BAPN-treated mice (Figure 2D-2H). From images of aortic tissue, we found that S100A4 overexpression significantly increased the degree and extent of hematoma in aortic dissection in BAPN-treated mice (Figure 2E). Moreover, EVG staining (Figure 2I) demonstrated that the overexpression of S100A4 aggravated BAPN-induced elastic fiber degradation (Figure 2J).

**Figure 3:**
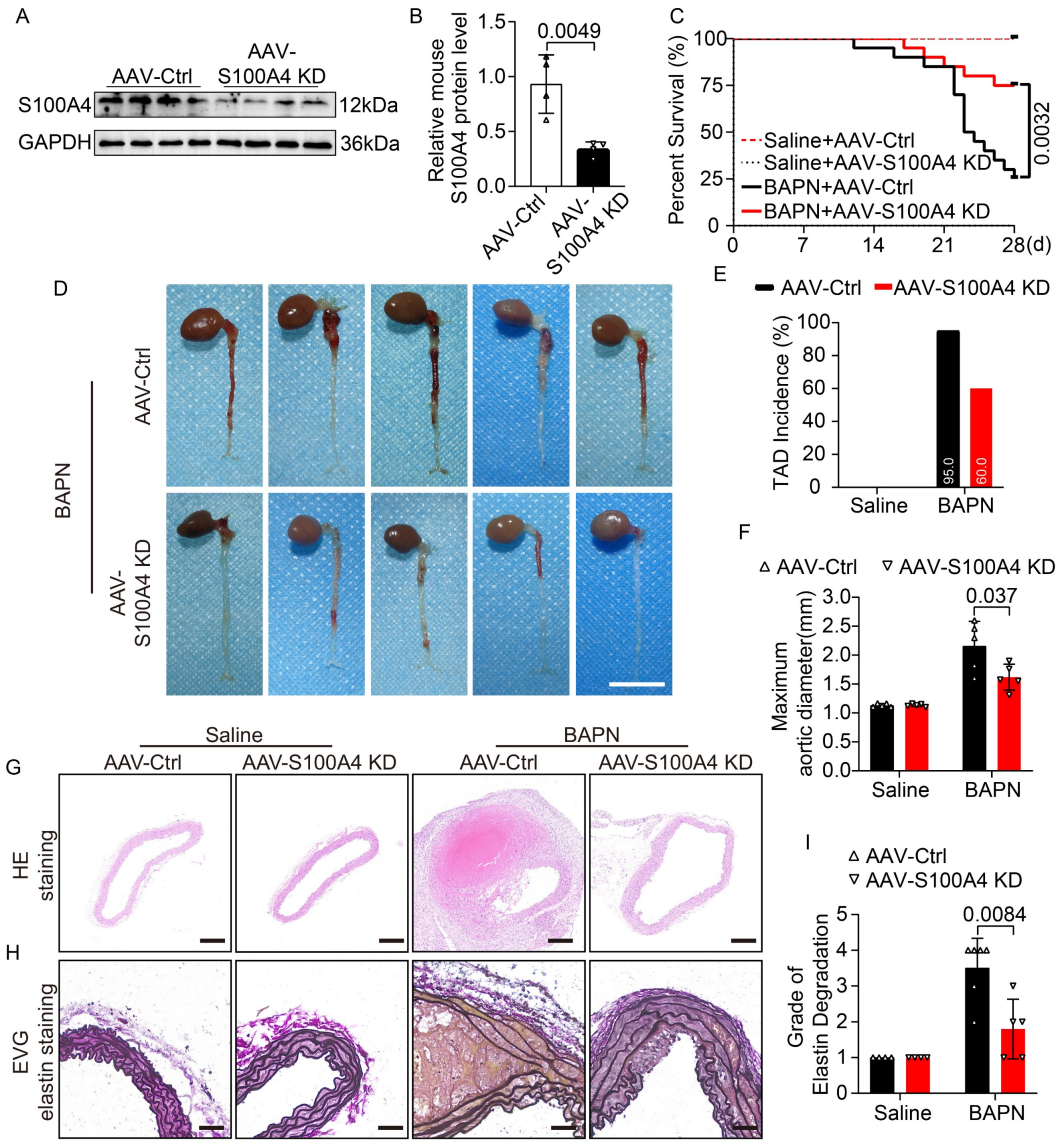
** Inhibition of S100A4 suppresses TAD formation and rupture.** Mice were injected with AAV9-Ctrl or AAV9-S100A4 KD intravenously at 18 days old and given 0.25% BAPN or equivalent saline for 4 weeks starting at 21 days of age (n=20 per group). **A-B,** Immunoblot analysis and quantification of S100A4 protein in aortic tissue from AAV9-Ctrl and AAV9-S100A4 KD mice (n=4). **C,** Survival curves in indicated groups (n=20 per group), log-rank test. **D,** Representative images of aortas. Scale bar: 1 cm. **E,** TAD incidence (n=20 per group). **F,** Measurements of maximum aortic diameter (n=4-6, normalized to body weight). **G,** Representative macroscopic images of ascending aorta sections stained with hematoxylin and eosin (H&E). Scale bars: 200 μm. **H,** Representative images of ascending aorta sections stained with Elastic-Van Gieson (EVG). Scale bars: 50 μm. **I,** Grade of elastin degradation in the aortic wall (n=4-6).

The functional role of S100A4 in aortic dissection was further validated in mice with AAV9-S100A4 knockdown (AAV9-S100A4 KD). First, we validated the efficient infection of AAV9-S100A4 KD and the expression of S100A4 in mouse arterial tissues (Figure 3A, 3B; Figure S3B). Subsequently, AAV-Ctrl and AAV-S100A4 KD mice were given 0.25% BAPN or equal amounts of normal saline in water for 4 weeks. We demonstrated that inhibition of S100A4 did not affect aortic dilation or TAD formation in mice treated with saline. Interestingly, inhibition of S100A4 reduced aortic dilation and TAD incidence in mice exposed to BAPN (Figure 3C-3F). In the analysis of aortic tissue images, we found that S100A4 inhibition significantly reduced the degree and extent of hematoma in aortic dissection in BAPN-treated mice (Figure 3D). Moreover, H&E (Figure 3G) and EVG (Figure 3H) staining showed that inhibition of S100A4 also mitigated BAPN-induced elastic fiber destruction (Figure 3I) and aortic lesions. These findings indicate the benefit of S100A4 inhibition on aortic tissue, ultimately inhibiting TAD formation.

### S100A4 exacerbates BAPN-induced phenotypic transformation of VSMCs

**Figure 4:**
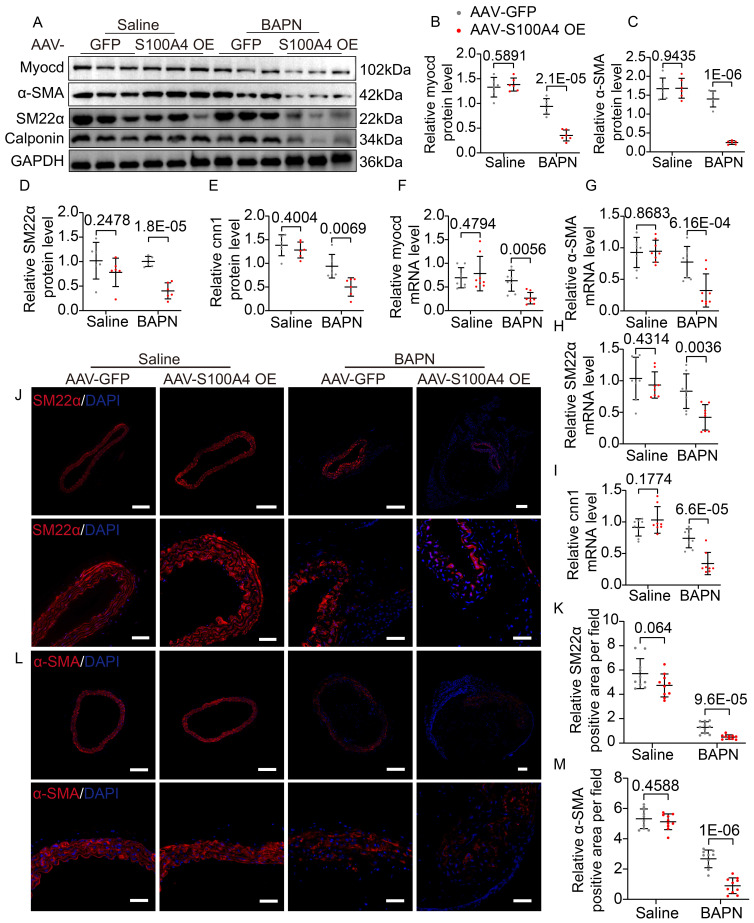
** Overexpression of S100A4 promotes BAPN-induced phenotypic transformation of VSMCs. A-E,** Representative immunoblotting and subsequent quantification of myocardin, α-SMA, SM22α, and calponin in the aortic wall from AAV9-GFP and AAV9-S100A4 OE mice (n = 6). **F-I,** RT-PCR analysis of myocardin, α-SMA, SM22α, and calponin in the aortic wall from AAV9-GFP and AAV9-S100A4 OE mice (n = 8). **J-K,** Representative immunofluorescence staining and subsequent quantification of SM22α in aortas from AAV9-GFP and AAV9-S100A4 OE mice. Scale bars: 200 μm (upper panel), 50 μm (lower panel). Ten fields of view were selected per mouse for calculation. The quantification of each image was normalized using DAPI. **L-M,** Representative immunofluorescence staining and subsequent quantification of α-SMA in aortas from AAV9-GFP and AAV9-S100A4 OE mice. Scale bars: 200 μm (upper panel), 50 μm (lower panel). Ten fields of view were selected per mouse for calculation. The quantification of each image was normalized using DAPI.

**Figure 5:**
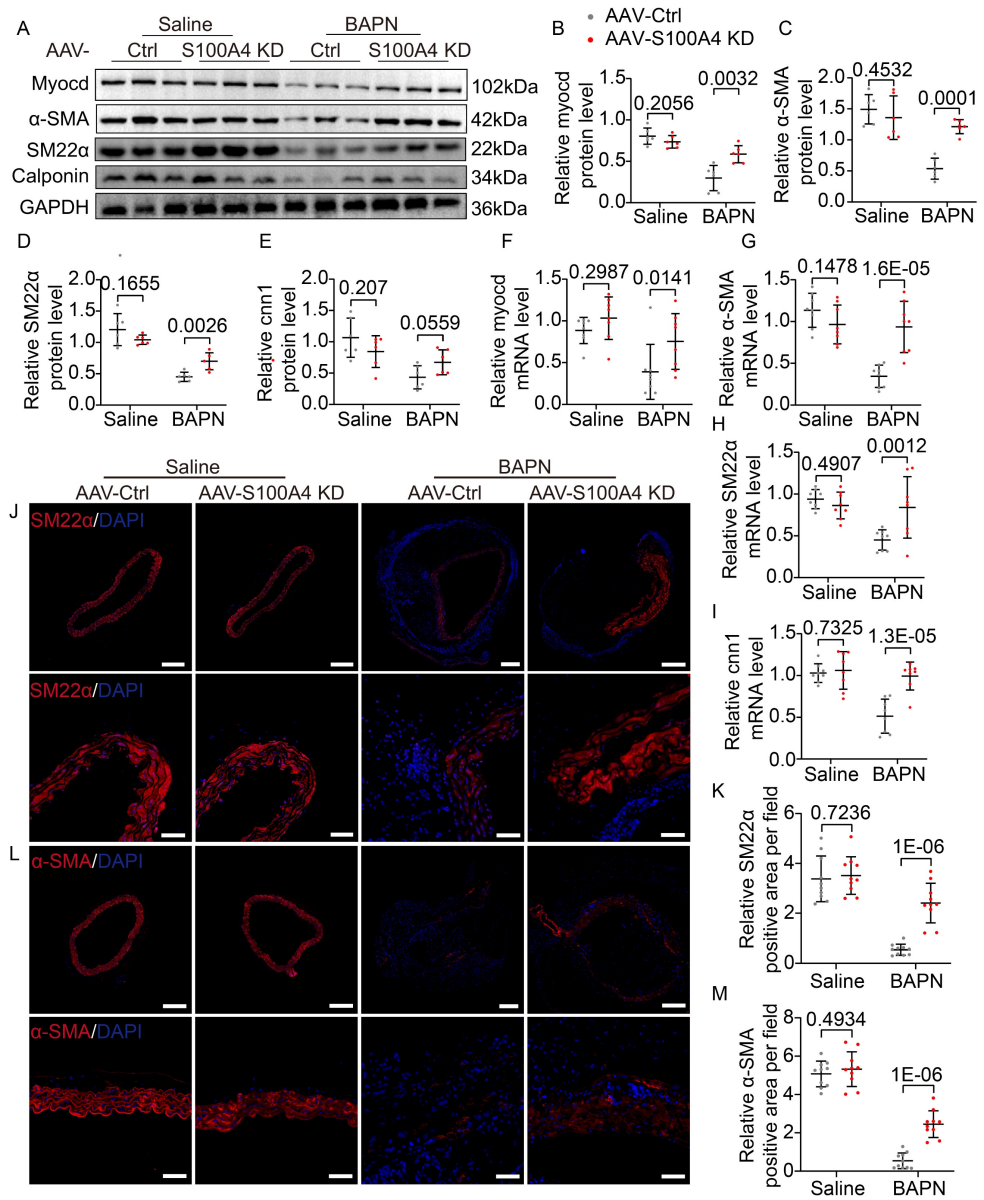
** Inhibition of S100A4 abolishes BAPN-induced phenotypic transformation of vascular smooth muscle cells (VSMCs). A-E,** Representative immunoblotting and subsequent quantification of myocardin, α-SMA, SM22α, and calponin in the aortic wall from AAV9-Crtl and AAV9-S100A4 KD mice (n = 6). **F-I,** RT-PCR analysis of myocardin, α-SMA, SM22α, and calponin in the aortic wall from AAV9-Crtl and AAV9-S100A4 KD mice (n = 8). **J-K,** Representative immunofluorescence staining and subsequent quantification of SM22α in aortas from AAV9-Crtl and AAV9-S100A4 KD mice. Scale bars: 200 μm (upper panel), 50 μm (lower panel). Ten fields of view were selected per mouse for calculation. The quantification of each image was normalized using DAPI. **L-M,** Representative immunofluorescence staining and subsequent quantification of α-SMA in aortas from AAV9-Crtl and AAV9-S100A4 KD mice. Scale bars: 200 μm (upper panel), 50 μm (lower panel). Ten fields of view were selected per mouse for calculation. The quantification of each image was normalized using DAPI.

Extracellular S100A4 causes phenotype switching in VSMCs 18,30, and the contractile phenotype of VSMCs is important for maintaining aortic wall homeostasis. Hence, we analyzed mouse aortic tissue to elucidate further the mechanism by which S100A4 aggravates TAD formation. WB (Figure 4A-4E), mRNA (Figure 4F-4I), and IF staining (Figure 4J-4M) analyses revealed that overexpression of S100A4 did not cause significant changes in contractile markers in the saline group. In contrast, overexpression of S100A4 exacerbated the reduction in contractile marker levels in BAPN-treated mice. The role of S100A4 in the VSMCs phenotype switch was further validated in AAV9-S100A4 KD mice. WB (Figure 5A-5E), mRNA (Figure 5F-5I), and IF staining (Figure 5J-5M) analyses revealed that contractile markers were significantly reduced in BAPN-treated AAV9-Ctrl mice and that the loss of S100A4 was able to mitigate the reduction of these contractile markers. Our data indicated that overexpression of S100A4 aggravated BAPN-induced phenotypic transformation of VSMCs, while conversely, loss of S100A4 mitigated the same transformation. This finding suggests that S100A4 may affect the formation and development of TAD through the phenotypic transition of VSMCs.

### S100A4 directly binds to LOX in VSMCs

**Figure 6:**
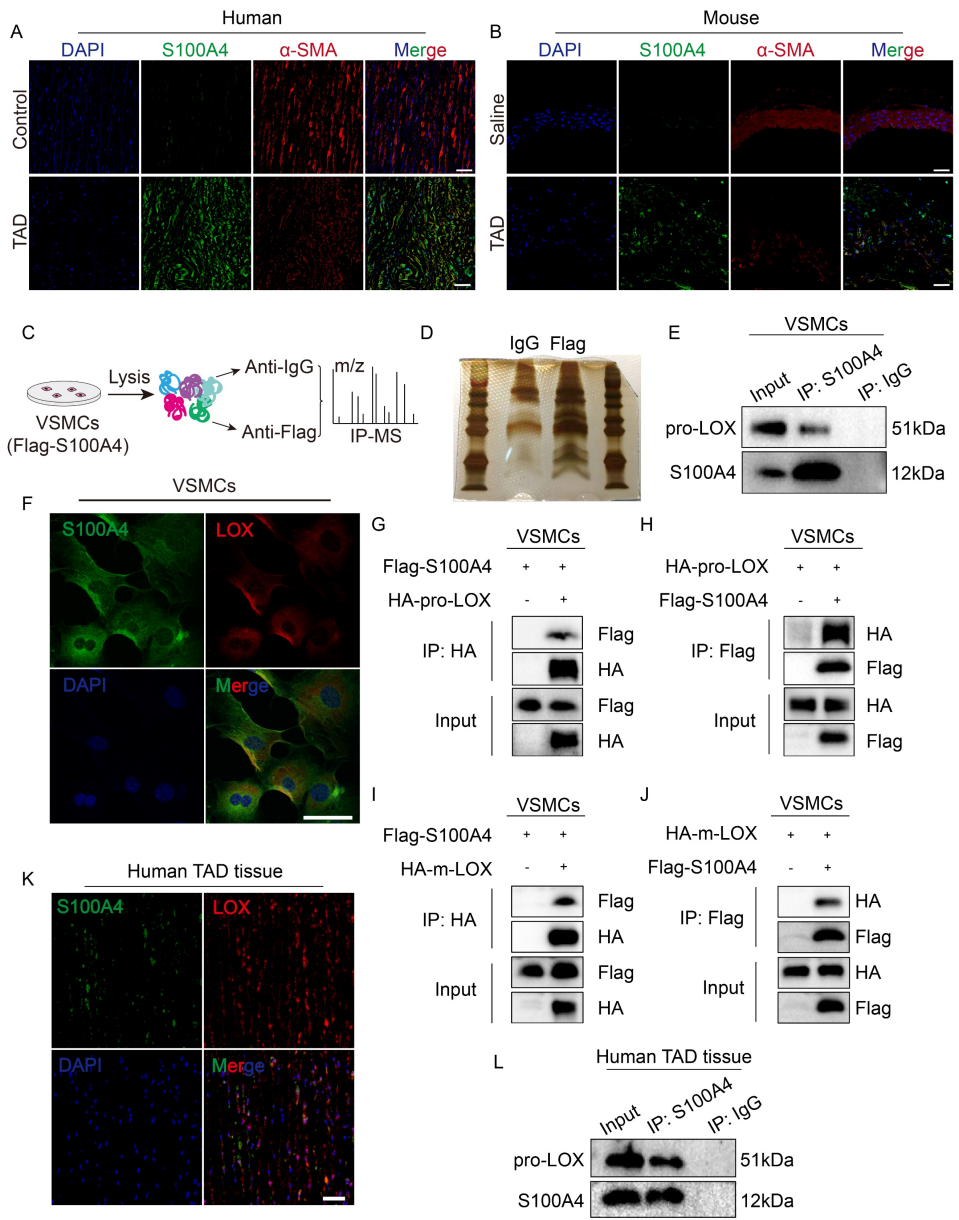
** Lysyl oxidase (LOX) was a potential downstream target of S100A4. A,** Representative confocal images of S100A4 colocalized with α-SMA in the aortic wall from control and patients with TAD. Scale bars: 50 μm. **B,** Representative confocal images of S100A4 colocalized with α-SMA in the aortic wall from control and TAD mice. Scale bars: 50 μm. **C,** Schematic diagram for screening specific protein interaction with S100A4 via mass spectrometry (MS) analysis. **D,** Silver staining image. **E,** Immunoprecipitation of FLAG (S100A4) and pro-LOX interactions in VSMCs. **F,** Immunostaining result show the co-localization of S100A4 and LOX in VSMCs. Scale bar: 50 μm. **G-J,** The indicated plasmids were transfected into VSMCs for 24 hours and co-immunoprecipitation and immunoblot analyses were performed. **K,** Representative confocal images of S100A4 co-localized with LOX in the aortic wall from control and patients with TAD. Scale bars: 50 μm. **L,** Immunoprecipitation analysis of S100A4 and pro-LOX interactions in the aortic wall from patients with TAD.

The mechanisms that drive the effect of extracellular S100A4 on VSMCs have been previously reported. However, the mechanisms for intracellular S100A4 have yet to be reported. According to previous reports 28, the expression of S100A4 increases in VSMCs after vascular injury, in correspondence with our findings (Figure 6A, 6B). To explore the specific mechanism of intracellular S100A4's formation and influence on TAD, we used IP-mass spectrometry analysis (Figure 6C, 6D) to identify key proteins interacting with S100A4. In the IP-mass spectrometry data, LOX belongs to the lysyl oxidase family, and the inactivation of the LOX gene causes structural changes in the aortic wall, resulting in TAD 31; thus, we speculated that LOX might be required for S100A4 function. Pro-LOX and S100A4 bands were present in the complex IP of the anti-S100A4 antibody; however, the control IgG was absent (Figure 6E). IF results showed that S100A4 and LOX were co-localized in mouse VSMCs (Figure 6F). Subsequently, we demonstrated that S100A4 and LOX interact in mouse VSMCs using exogenous IP assays (Figure 6G-6J). We also investigated the S100A4 and LOX in the aortic tissues from patients with TAD. IF results showed that S100A4 and LOX were co-localized in aortic tissues from patients with TAD (Figure 6K). Subsequently, we confirmed the interaction between S100A4 and pro-LOX in the aortic tissues from patients with TAD using IP (Figure 6L). Collectively, these results demonstrate that S100A4 binds LOX in VSMCs and aortic tissue.

### S100A4 inhibits elastic fiber deposition by reducing m-LOX

**Figure 7:**
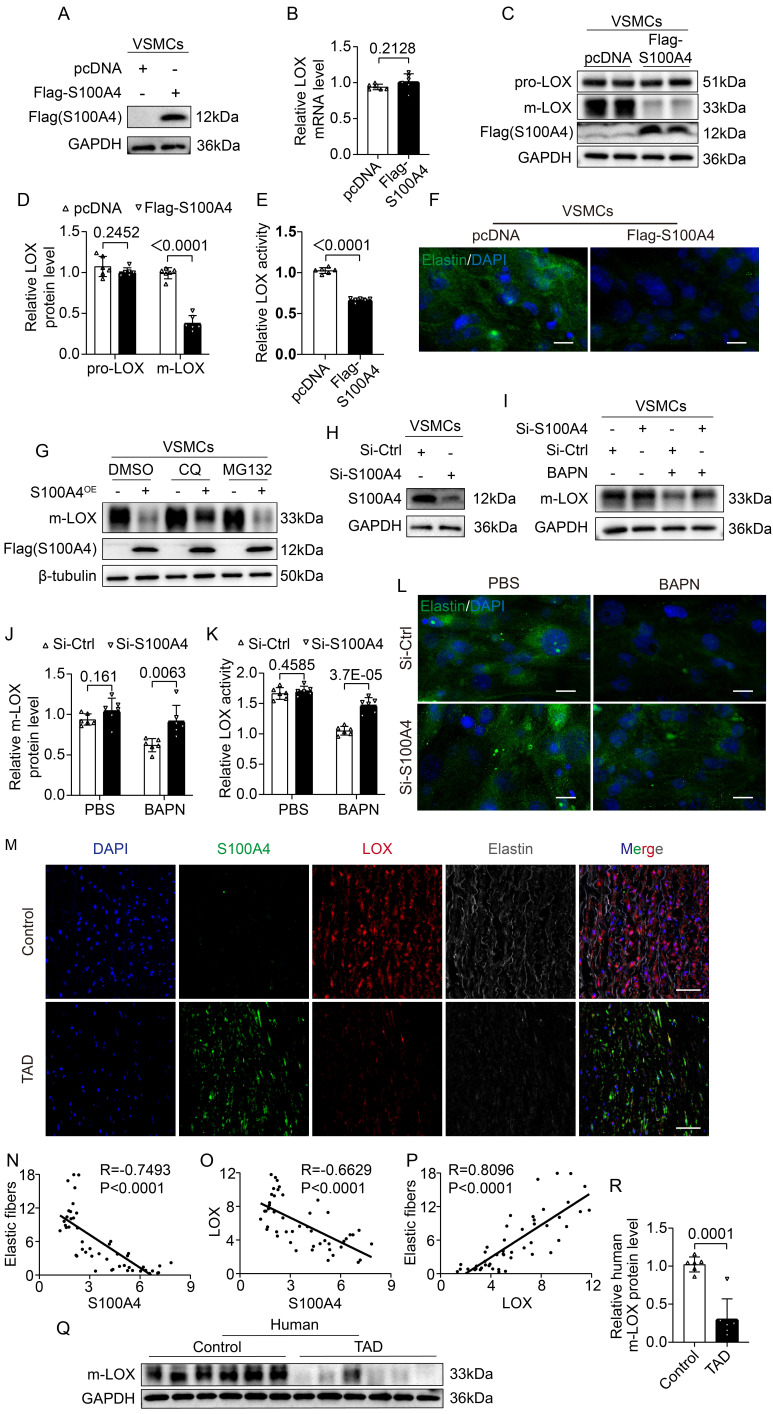
** S100A4 inhibits elastic fiber deposition by decreasing mature LOX (m-LOX) protein. A,** VSMCs transfected with the indicated plasmids, and immunoblot analysis of S100A4 overexpression in VSMCs. **B,** RT-PCR analysis of LOX in VSMCs transfected with the indicated plasmids 24 hours (n=6). **C-D,** Western blotting analysis and quantification of m-LOX and pro-LOX expression in VSMCs transfected with the indicated plasmids for 24 hours (n=6). **E,** Semi-quantitative analysis for LOX activity in the supernatant of VSMCs transfected with the indicated plasmids for 24 hours (n = 6). **F,** Immunostaining for elastin in VSMCs transfected with the indicated plasmids for 24 hours. Scale bars: 20 μm. **G,** Immunoblotting analysis of m-LOX protein expression in VSMCs transfected with the indicated plasmids for 24 hours and treated with dimethylsulfoxide (DMSO), chloroquine (CQ, 50 μM) or MG132 (50 μM) for 6 hours. **H,** The silencing effect of siRNA-S100A4 was verified via immunoblotting analysis. **I-J,** Immunoblotting analysis and quantification of m-LOX protein expression in VSMCs infected with control siRNA or S100A4 siRNA and treated with PBS or BAPN (250 ug/ml) for 24 hours (n = 6). **K,** Semi-quantitative analysis for LOX activity in the supernatant of VSMCs infected with control siRNA or S100A4 siRNA, treated with PBS or BAPN (250 ug/ml) for 24 hours (n = 6). **L,** Immunostaining for elastin in VSMCs infected with control siRNA or S100A4 siRNA and treated with PBS or BAPN (250 ug/ml) for 24 hours (n = 6). Scale bars: 20 μm. **M,** Representative images of S100A4, LOX, and elastin in human aortas from controls and patients with TAD. Scale bars: 50 μm. **N-P,** Quantification results of elastic fiber deposition and LOX and S100A4 protein expression using ImageJ, normalized with DAPI. R and P values were based on the correlation. **Q-R,** Immunoblotting analysis and quantification of m-LOX levels in aortic wall from controls and patients with TAD (n=6).

LOX becomes pre-protein (pro-LOX) following translation, and BMP-1 processes pro-LOX to yield the m-LOX form and its propeptide 32. Soluble elastin precursors are deposited on microfibers during the assembly of elastic fibers in the aortic wall. They are crosslinked by m-LOX into insoluble mature elastin called elastic fibers 33-35. Therefore, we investigated the effect of S100A4 on LOX expression. First, we validated a FLAG-tagged S100A4 plasmid (Figure 7A) and transfected the S100A4 plasmid in the VSMCs. Overexpression of S100A4 did not result in significant changes in transcription levels of LOX (Figure 7B). We found that increased expression of S100A4 did not result in significant changes in pro-LOX protein expression, but reduce the m-LOX protein levels in VSMCs (Figure 7C, 7D). Moreover, the m-LOX activity in the supernatant of VSMCs was significantly reduced (Figure 7E) and the elastin staining showed that the elastic fiber deposition was increased (Figure 7F). Interestingly, plasmids expressing HA-m-LOX effectively mitigated the deleterious alterations in elastic fiber deposition induced by S100A4 overexpression (Figure S4A). In support of this, we determined that lysosomal degradation inhibitors such as chloroquine could partially ameliorate the S100A4-induced reduction of m-LOX (Figure 7G). In comparison, proteasome inhibitor MG132 had no observable effect on the level of m-LOX reduction (Figure 7G). These data suggest that S100A4 degrades a portion of the m-LOX through the lysosomal pathway. To further determine the influence of S100A4 on m-LOX in VSMCs, we transfected and verified the knockdown efficiency of S100A4 siRNA (Figure 7H). Subsequently, we inhibited m-LOX by treating VSMCs with the LOX irreversible inhibitor BAPN. We found that S100A4 deficiency abrogated the BAPN-induced decrease in m-LOX in VSMCs (Figure 7I, 7J), resulting in increased secretion of m-LOX (Figure 7K) and deposition of more elastic fibers (Figure 7L).

We also investigated the expression and relationship of S100A4, LOX and elastic fiber deposition in human aortic tissue. When the expression of these three proteins was normalized, quantified, and counted using IF with DAPI, the expression of S100A4 was shown to be negatively correlated with the expression of LOX and the deposition of elastic fibers (Figure 7M-7O), while the expression of LOX was positively correlated with the deposition of elastic fibers (Figure 7P). Moreover, compared with the control group, the expression of protein S100A4 in TAD sample was significantly increased (Figure 1D-1I), whereas the expression of m-LOX was significantly decreased (Figure 7Q,7R).

**Figure 8:**
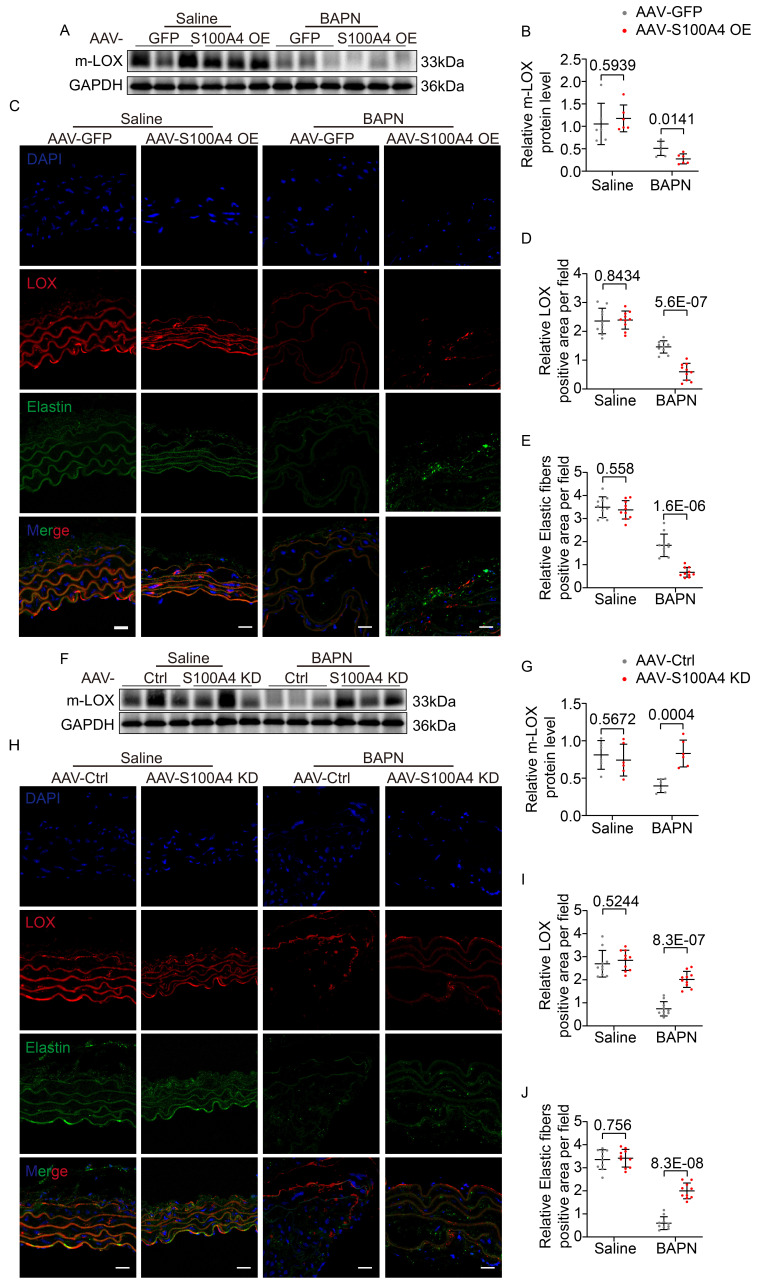
** S100A4 exacerbated BAPN-induced LOX reduction and decreased elastic fiber deposition. A-B,** Representative immunoblotting and subsequent quantification of m-LOX in the aortic wall from AAV9-GFP and AAV9-S100A4 OE mice (n = 6). **C-E,** Representative immunofluorescence staining and subsequent quantification of elastic fiber deposition and LOX in aortas from AAV9-GFP and AAV9-S100A4 OE mice. Scale bars: 20 μm. Ten fields of view were selected per mouse for calculation. The quantification of each image was normalized using DAPI. **F-G,** Representative immunoblotting and subsequent quantification of m-LOX in the aortic wall from AAV9-Ctrl and AAV9-S100A4 KD mice (n = 6). **H-J,** Representative immunofluorescence staining and subsequent quantification of elastic fiber deposition and LOX in aortas from AAV9-Ctrl and AAV9-S100A4 KD mice. Scale bars: 20 μm. Ten fields of view were selected per mouse for calculation. The quantification of each image was normalized using DAPI.

WB (Figure 8A, 8B) and IF staining (Figure 8C-8E) analyses revealed that AAV9-GFP mice exhibited slightly reduced m-LOX and elastic fiber deposition in the aorta after BAPN assimilation. In contrast, the overexpression of S100A4 further reduced m-LOX levels and mature elastic fiber deposition (Figure 8A-8E). Conversely, the inhibition of S100A4 had little effect on m-LOX expression and elastic fiber deposition in the saline group (Figure 8F-8J). However, following BAPN treatment, m-LOX expression and elastic fiber deposition in the aorta of AAV9-Ctrl mice were significantly decreased (Figure 8F-8J). In contrast, the inhibition of S100A4 rescued m-LOX and elastic fiber deposition levels (Figure 8F-8J). These unbiased findings suggest that S100A4 inhibits elastic fiber deposition by decreasing m-LOX.

### The expression of S100A4 has minimal impact on Collagen I (COL1) in the aorta and VSMCs

Collagen is the major component of the ECM of the aorta, providing strength against hemodynamic stress 36,37. Considering the ability of LOX also induces collagen crosslinking, we employed WB analysis and IF staining to detect COL1 expression in the aorta and VSMCs. Our result showed that COL1 expression in the aorta was reduced after BAPN treatment (Figure S5A-S5H). However, compared with the AAV-GFP group, the COL1 expression in the AAV-S100A4 OE group was slightly decreased, though the reduction was not significant (Figure S5A-S5D). Additionally, no significant difference was observed in the expression of COL1 between AAV-Ctrl and AAV-S100A4 KD mice (Figure S5E-S5H). After transfection of Flag-S100A4 expressing plasmid into VSMCs, the expression of COL1 was slightly decreased, though the reduction was not significant (Figure S5I-S5J). The results of this study showed that S100A4 had little effect on COL1, which is consistent with the results of previous studies 38,39.

## Discussion

To our knowledge, this study demonstrates for the first time that S100A4 can promote the occurrence and formation of TAD. This data, using TAD models in mice with overexpression and inhibition of S100A4, supports the potential value of S100A4 for therapeutic use in relation to TAD. In addition, our data indicate that S100A4 caused a decrease in active LOX by binding to LOX in VSMCs, causing a decrease in extracellular elastic fiber deposition. Moreover, we also found that S100A4 deficiency suppressed the VSMC phenotype switch, ultimately alleviating TAD development. These data suggest that S100A4 is critical to the formation and development of TAD and may provide effective therapeutic strategies for TAD.

S100A4 is a member of the S100 family. Many S100 family proteins, such as S100A8, S100A9, and S100A12, play a key role in thoracic aortic aneurysms and other cardiovascular diseases 40-42. Genetic ablation of S100A9 in Apo E^-/-^ mice alleviates the progression of atherosclerosis 40. Recent studies have shown that S100A4 is highly and significantly expressed in human carotid atherosclerotic plaques, and that the central part of the plaque has 2.1 times more expression of S100A4 than the surrounding stump area. Moreover, the expression S100A4 is positively correlated with the degree of expansive remodeling 43. This indicates that S100A4 has significance for vascular pathophysiology. To avoid sample interference in our experimental results, we excluded patients with TAD and atherosclerosis or hereditary aortic diseases such as Marfan syndrome when selecting aorta samples for the study 44. Other studies have shown that S100A4 deficiency improves cardiovascular diseases, including myocardial fibrosis, myocardial hypertrophy, and viral myocarditis 45,46. The absence of S100A4 may contribute to post-injury repair by alleviating the degradation of EMC components such as elastin 47. However, in a murine model of myocardial infarction, the ablation of S100A4 results in augmented tissue remodeling and fibrosis, ultimately exacerbating cardiac functional deterioration 48. In physiological states, the expression of S100A4 is either absent or very low 27. However, the expression of S100A4 is elevated under stress conditions or diseases, including pathological myocardial hypertrophy, myocardial ischemia, and pulmonary hypertension 28,29,45,49. Similarly, our data showed that S100A4 was significantly increased in the aorta samples of both humans and mice following TAD onset, in agreement with previous studies.

Under physiological conditions, VSMC maintains the homeostasis of ECM in blood vessels. But under pathological conditions, VSMC transitions from a contractile phenotype that maintains a physiological state to a pathogenic synthetic and pro-inflammatory phenotype, a fundamental factor causing ECM degradation, aortic wall weakening, and thoracic aortic aneurysm (TAA) rupture 50,51. The expression of S100A4 increases in stimulated or synthetic VSMCs 27,28. Treatment of VSMCs with recombinant oligomeric S100A4 induces nuclear factor kappa-light-chain-enhancer of activated B cells (NF-κB) and changes VSMCs to a pro-inflammatory phenotype. In contrast, the neutralization of extracellular S100A4 promotes atherosclerotic plaque stabilization 18. In our study, S100A4 deletion significantly increased the expression of contractile markers, which mitigated TAD progression. Our findings agree with previous research showing that the phenotypic switching of VSMCs is involved in dissection and aneurysm progression. Objectively, although we tried to remove the media for the mouse aortic tissues used for WBs and RT-PCR, it is important to consider the inevitable contamination of the adventitia and endothelium.

The disruption of ECM homeostasis is one of the most important components of aortic dissection 1,9. During aorta development, once blood flow occurs, VSMCs begin to produce ECM 33, which not only provides structural support for the main artery under physiological conditions but also affects the pathophysiological process of blood vessels 52. Importantly, elastic fibers are the largest and most stable structures within ECM. Once the elastic fibers in ECM are destroyed, homeostasis is disrupted, which leads to the development of vascular diseases 53-55. During the formation of elastic fibers, VSMCs, endothelial cells, and fibroblasts secrete soluble precursor tropoelastin, which is deposited on microfiber scaffolders 9,10,56,57.

Adjacent tropoelastin proteins spontaneously aggregate, then crosslink through the copper-dependent family enzymes of LOX to form insoluble elastin polymers called elastic fibers 9,10, the functional form of the mature protein. In the present study, we observed that deletion of S100A4 significantly improved the elastic fibers deposition, thereby maintaining ECM homeostasis and slowing TAD development.

LOX is a copper-dependent amine oxidase that catalyzes the covalent crosslinking of elastin and collagen and maintains ECM homeostasis 58. Moreover, 80% of LOX activity in aortic smooth muscle cells is the LOX isoform 59; thus, LOX is essential for maintaining aortic vascular wall tension and elasticity. Loss, inhibition, or mutation of LOX causes spontaneous coronary dissection, TAA, and TAD 1,31,60. LOX becomes pro-LOX after undergoing post-translational modification, and pro-LOX is subsequently processed by BMP-1 into active m-LOX and its propeptide 32. The transcriptional regulatory mechanism of LOX has been widely studied. On the one hand, transforming growth factor-β (TGF-β), platelet derived growth factor (PDGF), and angiotensin II (Ang II) all induce the expression of the LOX gene in vascular tissue 61. On the other hand, tumor necrosis factor-alpha (TNF-α), low-density lipoproteins (LDL), interferon-gamma (INF-γ), and high levels of homocysteine decrease the expression of the LOX gene in vascular cells, including VSMCs 62-65. Many studies have reported the mechanism of LOX transcriptional regulation; however, our understanding of LOX protein metabolism is limited. A previous study has shown that the LOX protein is degraded by lysosomes in Purkinje cells 66. Prostaglandin E2-receptor EP4 (PGE2-EP4) signaling promotes lysosomal degradation of m-LOX protein in ductus arteriosus 67. Coincidentally, we demonstrated for the first time that S100A4 binds to LOX and causes a reduction in the level of LOX, which is partially degraded by the lysosomal pathway, in correspondence with previous research.

This study has some limitations. First, the effect of S100A4 on TAD via LOX requires further study in alternative TAD mouse models to exclude the effect of BAPN on the specific inhibition of LOX. Second, our finding suggest that S100A4 disrupts ECM homeostasis and VSMC phenotype. However, the relationship between ECM homeostasis and VSMC phenotype and determining which factor is more important for the formation and development of TAD requires further investigation of the specific mechanism of TAD formation.

In conclusion, our research elucidates for the first time the influence that S100A4 has on TAD development. While TAD onset, the expression of S100A4 is increased, accompanied by the phenotypic transition of VSMCs and the decrease of elastic fiber deposition in ECM. In addition, S100A4 directly interacts with LOX to promote m-LOX lysosomal degradation, thereby reducing elastic fiber deposition and inducing vascular degeneration and TAD progression. S100A4-targeted therapy may provide non-surgical therapeutic options for TAD, a disease frequently resulting in fatal outcomes.

## Supplementary Material

Supplementary methods, figures and tables.

## Figures and Tables

**Figure 9:**
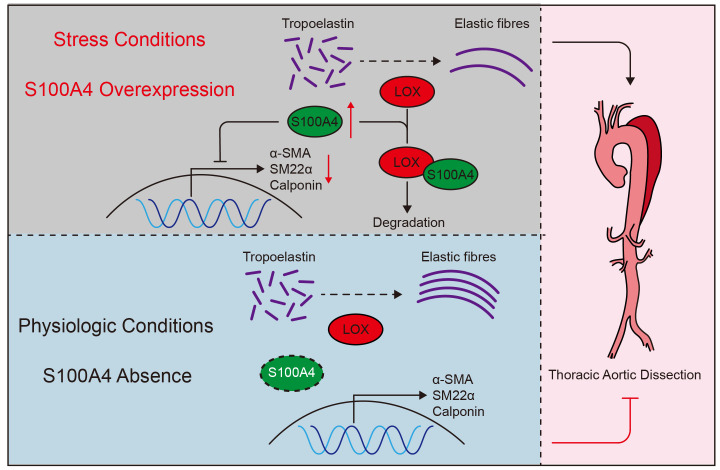
** Schematic summary.** Under stressful conditions, overexpression of S100A4 promotes VSMCs phenotype transition, LOX degradation, and elastin fiber deposition, exacerbating the formation and development of TAD. Under physiological conditions, S100A4 expression is absent. These results suggest that S100A4 is essential for the formation and development of TAD and may provide an effective treatment strategy for TAD.

## Abbreviations

TAD: Thoracic aortic dissection
VSMCs: vascular smooth muscle cells
ECM: extracellular matrix
BAPN: β-aminopropionitrile monofumarate
DMEM: Dulbecco's Modified Eagle Medium
siRNA: Small interfering RNA
H&E: hematoxylin and Eosin
EVG: Elastic Van Gieson
α-SMA: alpha-smooth muscle actin
SM22α: smooth muscle protein 22-α
PBS: phosphate-buffered saline
GAPDH: glyceraldehyde-3-phosphate dehydrogenase
PCR: polymerase chain reaction
qPCR: quantitative polymerase chain reaction
co-IP: co-immunoprecipitation
TAA: thoracic aortic aneurysm

## References

[1] Nienaber CA, Clough RE, Sakalihasan N, Suzuki T, Gibbs R, Mussa F (2016). Aortic dissection. Nat Rev Dis Primers.

[2] Mussa FF, Horton JD, Moridzadeh R, Nicholson J, Trimarchi S, Eagle KA (2016). Acute Aortic Dissection and Intramural Hematoma: A Systematic Review. Jama.

[3] Guo DC, Papke CL, He R, Milewicz DM (2006). Pathogenesis of thoracic and abdominal aortic aneurysms. Ann N Y Acad Sci.

[4] Owens GK, Kumar MS, Wamhoff BR (2004). Molecular regulation of vascular smooth muscle cell differentiation in development and disease. Physiol Rev.

[5] Nogi M, Satoh K, Sunamura S, Kikuchi N, Satoh T, Kurosawa R (2018). Small GTP-Binding Protein GDP Dissociation Stimulator Prevents Thoracic Aortic Aneurysm Formation and Rupture by Phenotypic Preservation of Aortic Smooth Muscle Cells. Circulation.

[6] Yang K, Ren J, Li X, Wang Z, Xue L, Cui S (2020). Prevention of aortic dissection and aneurysm via an ALDH2-mediated switch in vascular smooth muscle cell phenotype. Eur Heart J.

[7] Toyohara T, Roudnicky F, Florido MHC, Nakano T, Yu H, Katsuki S (2020). Patient hiPSCs Identify Vascular Smooth Muscle Arylacetamide Deacetylase as Protective against Atherosclerosis. Cell Stem Cell.

[8] Saboor F, Reckmann AN, Tomczyk CU, Peters DM, Weissmann N, Kaschtanow A (2016). Nestin-expressing vascular wall cells drive development of pulmonary hypertension. Eur Respir J.

[9] Kielty CM, Sherratt MJ, Shuttleworth CA (2002). Elastic fibres. J Cell Sci.

[10] Kielty CM (2006). Elastic fibres in health and disease. Expert Rev Mol Med.

[11] Garrett SC, Varney KM, Weber DJ, Bresnick AR (2006). S100A4, a mediator of metastasis. J Biol Chem.

[12] Gonzalez LL, Garrie K, Turner MD (2020). Role of S100 proteins in health and disease. Biochim Biophys Acta Mol Cell Res.

[13] Bresnick AR, Weber DJ, Zimmer DB (2015). S100 proteins in cancer. Nat Rev Cancer.

[14] Schmidt-Hansen B, Ornås D, Grigorian M, Klingelhöfer J, Tulchinsky E, Lukanidin E (2004). Extracellular S100A4(mts1) stimulates invasive growth of mouse endothelial cells and modulates MMP-13 matrix metalloproteinase activity. Oncogene.

[15] Piera-Velazquez S, Jimenez SA (2019). Endothelial to Mesenchymal Transition: Role in Physiology and in the Pathogenesis of Human Diseases. Physiol Rev.

[16] Bettum IJ, Vasiliauskaite K, Nygaard V, Clancy T, Pettersen SJ, Tenstad E (2014). Metastasis-associated protein S100A4 induces a network of inflammatory cytokines that activate stromal cells to acquire pro-tumorigenic properties. Cancer Lett.

[17] Hansen MT, Forst B, Cremers N, Quagliata L, Ambartsumian N, Grum-Schwensen B (2015). A link between inflammation and metastasis: serum amyloid A1 and A3 induce metastasis, and are targets of metastasis-inducing S100A4. Oncogene.

[18] Sakic A, Chaabane C, Ambartsumian N, Klingelhöfer J, Lemeille S, Kwak BR (2022). Neutralization of S100A4 induces stabilization of atherosclerotic plaques: role of smooth muscle cells. Cardiovasc Res.

[19] Zhang X, Yang Z, Li X, Liu X, Wang X, Qiu T (2022). Bioinformatics Analysis Reveals Cell Cycle-Related Gene Upregulation in Ascending Aortic Tissues From Murine Models. Front Genet.

[20] Yang H, Zhou T, Stranz A, DeRoo E, Liu B (2021). Single-Cell RNA Sequencing Reveals Heterogeneity of Vascular Cells in Early Stage Murine Abdominal Aortic Aneurysm-Brief Report. Arterioscler Thromb Vasc Biol.

[21] Cao J, Geng L, Wu Q, Wang W, Chen Q, Lu L (2013). Spatiotemporal expression of matrix metalloproteinases (MMPs) is regulated by the Ca2+-signal transducer S100A4 in the pathogenesis of thoracic aortic aneurysm. PLoS One.

[22] Chen JY, Tsai PJ, Tai HC, Tsai RL, Chang YT, Wang MC (2013). Increased aortic stiffness and attenuated lysyl oxidase activity in obesity. Arterioscler Thromb Vasc Biol.

[23] Cui H, Chen Y, Li K, Zhan R, Zhao M, Xu Y (2021). Untargeted metabolomics identifies succinate as a biomarker and therapeutic target in aortic aneurysm and dissection. Eur Heart J.

[24] Lelièvre E, Hinek A, Lupu F, Buquet C, Soncin F, Mattot V (2008). VE-statin/egfl7 regulates vascular elastogenesis by interacting with lysyl oxidases. Embo j.

[25] Satoh K, Nigro P, Matoba T, O'Dell MR, Cui Z, Shi X (2009). Cyclophilin A enhances vascular oxidative stress and the development of angiotensin II-induced aortic aneurysms. Nat Med.

[26] Kitamoto S, Sukhova GK, Sun J, Yang M, Libby P, Love V (2007). Cathepsin L deficiency reduces diet-induced atherosclerosis in low-density lipoprotein receptor-knockout mice. Circulation.

[27] Brisset AC, Hao H, Camenzind E, Bacchetta M, Geinoz A, Sanchez JC (2007). Intimal smooth muscle cells of porcine and human coronary artery express S100A4, a marker of the rhomboid phenotype i*n vitro*. Circ Res.

[28] Furmanik M, Chatrou M, van Gorp R, Akbulut A, Willems B, Schmidt H (2020). Reactive Oxygen-Forming Nox5 Links Vascular Smooth Muscle Cell Phenotypic Switching and Extracellular Vesicle-Mediated Vascular Calcification. Circ Res.

[29] Lawrie A, Spiekerkoetter E, Martinez EC, Ambartsumian N, Sheward WJ, MacLean MR (2005). Interdependent serotonin transporter and receptor pathways regulate S100A4/Mts1, a gene associated with pulmonary vascular disease. Circ Res.

[30] Chaabane C, Heizmann CW, Bochaton-Piallat ML (2015). Extracellular S100A4 induces smooth muscle cell phenotypic transition mediated by RAGE. Biochim Biophys Acta.

[31] Guo DC, Regalado ES, Gong L, Duan X, Santos-Cortez RL, Arnaud P (2016). LOX Mutations Predispose to Thoracic Aortic Aneurysms and Dissections. Circ Res.

[32] Rodríguez C, Martínez-González J, Raposo B, Alcudia JF, Guadall A, Badimon L (2008). Regulation of lysyl oxidase in vascular cells: lysyl oxidase as a new player in cardiovascular diseases. Cardiovasc Res.

[33] Wagenseil JE, Mecham RP (2009). Vascular extracellular matrix and arterial mechanics. Physiol Rev.

[34] Nakamura T, Lozano PR, Ikeda Y, Iwanaga Y, Hinek A, Minamisawa S (2002). Fibulin-5/DANCE is essential for elastogenesis *in vivo*. Nature.

[35] Al-U'datt D, Allen BG, Nattel S (2019). Role of the lysyl oxidase enzyme family in cardiac function and disease. Cardiovasc Res.

[36] Quintana RA, Taylor WR (2019). Cellular Mechanisms of Aortic Aneurysm Formation. Circ Res.

[37] Chou E, Pirruccello JP, Ellinor PT, Lindsay ME (2023). Genetics and mechanisms of thoracic aortic disease. Nat Rev Cardiol.

[38] Hornstra IK, Birge S, Starcher B, Bailey AJ, Mecham RP, Shapiro SD (2003). Lysyl oxidase is required for vascular and diaphragmatic development in mice. J Biol Chem.

[39] Mäki JM, Räsänen J, Tikkanen H, Sormunen R, Mäkikallio K, Kivirikko KI (2002). Inactivation of the lysyl oxidase gene Lox leads to aortic aneurysms, cardiovascular dysfunction, and perinatal death in mice. Circulation.

[40] Hofmann Bowman M, Wilk J, Heydemann A, Kim G, Rehman J, Lodato JA (2010). S100A12 mediates aortic wall remodeling and aortic aneurysm. Circ Res.

[41] Averill MM, Kerkhoff C, Bornfeldt KE (2012). S100A8 and S100A9 in cardiovascular biology and disease. Arterioscler Thromb Vasc Biol.

[42] Oesterle A, Bowman MA (2015). S100A12 and the S100/Calgranulins: Emerging Biomarkers for Atherosclerosis and Possibly Therapeutic Targets. Arterioscler Thromb Vasc Biol.

[43] Nagata M, Minami M, Yoshida K, Yang T, Yamamoto Y, Takayama N (2020). Calcium-Binding Protein S100A4 Is Upregulated in Carotid Atherosclerotic Plaques and Contributes to Expansive Remodeling. J Am Heart Assoc.

[44] LeMaire SA, Russell L (2011). Epidemiology of thoracic aortic dissection. Nat Rev Cardiol.

[45] Tamaki Y, Iwanaga Y, Niizuma S, Kawashima T, Kato T, Inuzuka Y (2013). Metastasis-associated protein, S100A4 mediates cardiac fibrosis potentially through the modulation of p53 in cardiac fibroblasts. J Mol Cell Cardiol.

[46] Wang W, Ma K, Liu J, Li F (2019). Ginkgo biloba extract may alleviate viral myocarditis by suppression of S100A4 and MMP-3. J Med Virol.

[47] Kim YM, Haghighat L, Spiekerkoetter E, Sawada H, Alvira CM, Wang L (2011). Neutrophil elastase is produced by pulmonary artery smooth muscle cells and is linked to neointimal lesions. Am J Pathol.

[48] Doroudgar S, Quijada P, Konstandin M, Ilves K, Broughton K, Khalafalla FG (2016). S100A4 protects the myocardium against ischemic stress. J Mol Cell Cardiol.

[49] Schneider M, Kostin S, Strøm CC, Aplin M, Lyngbaek S, Theilade J (2007). S100A4 is upregulated in injured myocardium and promotes growth and survival of cardiac myocytes. Cardiovasc Res.

[50] Bennett MR, Sinha S, Owens GK (2016). Vascular Smooth Muscle Cells in Atherosclerosis. Circ Res.

[51] Liu R, Lo L, Lay AJ, Zhao Y, Ting KK, Robertson EN (2017). ARHGAP18 Protects Against Thoracic Aortic Aneurysm Formation by Mitigating the Synthetic and Proinflammatory Smooth Muscle Cell Phenotype. Circ Res.

[52] Hynes RO (2009). The extracellular matrix: not just pretty fibrils. Science.

[53] Galis ZS, Sukhova GK, Lark MW, Libby P (1994). Increased expression of matrix metalloproteinases and matrix degrading activity in vulnerable regions of human atherosclerotic plaques. J Clin Invest.

[54] Katsuda S, Okada Y, Okada Y, Imai K, Nakanishi I (1994). Matrix metalloproteinase-9 (92-kd gelatinase/type IV collagenase equals gelatinase B) can degrade arterial elastin. Am J Pathol.

[55] Li DY, Brooke B, Davis EC, Mecham RP, Sorensen LK, Boak BB (1998). Elastin is an essential determinant of arterial morphogenesis. Nature.

[56] Mithieux SM, Weiss AS (2005). Elastin. Adv Protein Chem.

[57] Hinek A, Rabinovitch M (1994). 67-kD elastin-binding protein is a protective "companion" of extracellular insoluble elastin and intracellular tropoelastin. J Cell Biol.

[58] Kagan HM, Li W (2003). Lysyl oxidase: properties, specificity, and biological roles inside and outside of the cell. J Cell Biochem.

[59] Mäki JM, Sormunen R, Lippo S, Kaarteenaho-Wiik R, Soininen R, Myllyharju J (2005). Lysyl oxidase is essential for normal development and function of the respiratory system and for the integrity of elastic and collagen fibers in various tissues. Am J Pathol.

[60] Sibon I, Sommer P, Lamaziere JM, Bonnet J (2005). Lysyl oxidase deficiency: a new cause of human arterial dissection. Heart.

[61] Yoshimura K, Aoki H, Ikeda Y, Fujii K, Akiyama N, Furutani A (2005). Regression of abdominal aortic aneurysm by inhibition of c-Jun N-terminal kinase. Nat Med.

[62] Rodríguez C, Alcudia JF, Martínez-González J, Raposo B, Navarro MA, Badimon L (2008). Lysyl oxidase (LOX) down-regulation by TNFalpha: a new mechanism underlying TNFalpha-induced endothelial dysfunction. Atherosclerosis.

[63] Rodríguez C, Raposo B, Martínez-González J, Casaní L, Badimon L (2002). Low density lipoproteins downregulate lysyl oxidase in vascular endothelial cells and the arterial wall. Arterioscler Thromb Vasc Biol.

[64] Song YL, Ford JW, Gordon D, Shanley CJ (2000). Regulation of lysyl oxidase by interferon-gamma in rat aortic smooth muscle cells. Arterioscler Thromb Vasc Biol.

[65] Raposo B, Rodríguez C, Martínez-González J, Badimon L (2004). High levels of homocysteine inhibit lysyl oxidase (LOX) and downregulate LOX expression in vascular endothelial cells. Atherosclerosis.

[66] Peng C, Yan S, Ye J, Shen L, Xu T, Tao W (2012). Vps18 deficiency inhibits dendritogenesis in Purkinje cells by blocking the lysosomal degradation of Lysyl Oxidase. Biochem Biophys Res Commun.

[67] Yokoyama U, Minamisawa S, Shioda A, Ishiwata R, Jin MH, Masuda M (2014). Prostaglandin E2 inhibits elastogenesis in the ductus arteriosus via EP4 signaling. Circulation.

