# Supplementary material for: S100A4 Is a Key Facilitator of Thoracic Aortic Dissection
Source: Int J Biol Sci. 2024 Jan 1;20(1):29–46. doi: 10.7150/ijbs.83091 (PMC10750273; doi:10.7150/ijbs.83091)
Supplement: Supplementary file 1 — Supplementary methods, figures and tables. [file ijbsv20p0029s1.pdf]

SUPPLEMENTAL MATERIALS

S100A4 Is a Key Facilitator of Thoracic Aortic Dissection

Jiajun Shi[1#], Wenjun Yu[1,2,3#], Chuan Liang[1,2,3], Hongjie Shi[1], Dengwei Cao[1], Yong Ran[1], Haisen Qiao[1], Zhe Dong[1,2,3*], Jinping Liu[1,2,3*]

Affiliations

[1]Department of Cardiovascular Surgery, Zhongnan Hospital of Wuhan University, Wuhan 430071, China;

[2]Hubei Provincial Engineering Research Center of Minimally Invasive Cardiovascular Surgery, Wuhan 430071, China;

[3]Wuhan Clinical Research Center for Minimally Invasive Treatment of Structural Heart Disease, Wuhan 430071, China;

SUPPLEMENTAL METHODS

Data collection and process

RNA-sequencing data of aortic dissection mouse were obtained from the Gene Expression Omnibus (GEO; http://www.ncbi.nlm.nih.gov/geo). Four data sets from GPL10787 platform (GSE107479, normal: n=8, AD: n=5; GSE116434, normal: n=3, AD: n=3; GSE138484, normal: n=3, AD: n=3; GSE138558, normal: n=3, AD: n=3) were merged and remove batch effect using “sva” R package. tSNE analysis suggested that the batch effect was eliminated successfully (Figure S1A).

Weighted Gene Co-Expression Network Analysis (WGCNA)

“WGCNA” algorithm was used to construct the co-expression network based on the top 25% variant genes. Sample dendrogram and trait indicator was drawn after detecting outliers (Figure S1B). Pearson's correlation matrices were used for all pair-wise genes and a weighted adjacency matrix was constructed subsequently. Then a scale-free network, which was used for penalizing weak correlations and emphasizing strong correlations, was built based on $\beta=3$ (scale-free $R2 = 0.9$) (Figure S1C). The topology overlay matrix (TOM), which was converted from the adjacency matrix, computed the network connectivity of genes and generate 10 gene modules finally (Figure S1D).

Least Absolute Shrinkage and Selection Operator (LASSO) and Support Vector Machine-Recursive Feature Elimination (SVM-RFE)

Algorithms

Genes selected using “WGCNA” algorithm were respectively analyzed by LASSO and SVM-RFE algorithms for hub features selection. The glmnet package could performe the LASSO analysis. By removing SVM-generated eigenvectors, the machine learning method “SVM-RFE” based on support vector machine could find the best features (Figure S1E).

Gene Ontology enrichment analysis

We divided aortic dissection mice into a high-S100A4 expression and low-S100A4 expression groups according to the median expression value of S100A4, and used the "Limma" package for differential analysis ($|LogFC| \geqslant 0.58$, $p<0.05$). Subsequently, the up-regulated and down-regulated genes in the high S100A4 group were imported into the Metascape online database (https://metascape.org/gp/index.html#/main/step1) for enrichment analysis, respectively.

SUPPLEMENTAL FIGURE

Figure S1. Selection of the hub genes in AD.

A. tSNE plot before (left) and after (right) removing batch effects. B. Sample dendrogram and trait indicator. The color intensity was proportional to sample information. C. Determination of soft threshold power in WGCNA (scale-free R2 = 0.9). β = 3 was chosen as the optimal soft-thresholding parameter. D. Heatmap of the correlation between sample information and gene modules (pearson analysis). E. Feature selection based LASSO and SVM-RFE algorithms.

Figure S2. Gene Ontology enrichment analysis

A-B, Gene ontology enrichment analysis based on differentially expressed genes between high and low S100A4 expression group (|LogFC|⩾0.58, p<0.05).

Figure S3. Expression of AAV9-S100A4 in the ascending aorta of mice

A. Representative immunofluorescence staining of S100A4 and α-SMA in ascending aortas from AAV9-GFP and AAV9-S100A4 OE mice. Scale bars: 50 μm.

A. Representative immunofluorescence staining of S100A4 and α-SMA in ascending aortas from AAV9-Ctrl and AAV9-S100A4 KD mice. Scale bars: 50 μm.

Figure S4. Mature-LOX (m-LOX) improves elastic fiber deposition

A, Immunostaining for elastin in VSMCs transfected with the indicated plasmids for 24 hours. Scale bars: 20 μm.

Figure S5. Collagen I (COL1) is minimally affected in aortas and VSMCs with altered S100A4 expression

A-B, Representative immunoblotting and subsequent quantification of COL1 in aortic wall from AAV9-GFP and AAV9-S100A4 OE mice (n = 6). **C-D,** Representative immunofluorescence staining and subsequent quantification of COL1 in aortas from AAV9-GFP and AAV9-S100A4 OE mice. Scale bars: 50 μm. Ten fields of view were selected per mouse for calculation. The quantification of each image was normalized using DAPI. **E-F,** Representative immunoblotting and subsequent quantification of COL1 in aortic wall from AAV9-Crtl and AAV9-S100A4 KD mice (n = 6). **G-H,** Representative immunofluorescence staining and subsequent quantification of COL1 in aortas from AAV9-Crtl and AAV9-S100A4 KD mice. Scale bars: 50 μm. Ten fields of view were selected per mouse for calculation. The quantification of each image was normalized using DAPI.

SUPPLEMENTAL TABLE

Table S1. Patient Characteristics

Characteristics	Controls (n=6)	TAD (n=10)
Age (y)	55.4±9.8	57.1±9.1
Male	4 (66.7%)	7 (70%)
Coronary artery disease	0 (0%)	0 (0%)
Hyperlipidemia	0 (0%)	0 (0%)
Marfan syndrome	0 (0%)	0 (0%)
Diabetes mellitus	0 (0%)	1 (10%)
Hypertension	3 (50%)	8 (80%)
History of smoking	1 (16.7%)	3 (30%)
Aortic diameter (cm)	NA	5.5±1

Data are expressed as a number (percent) or as the mean ± standard deviation. TAD: thoracic aortic tissue from patients with acute ascending thoracic aortic dissection.

Table S2. Animals (in vivo studies)

Species	Vendor or Source	Background Strain	Sex
Mouse	Beijing Vital River Laboratory Animal Technology Co., Ltd.	C57BL/6J	Male

Table S3. Antibodies

Target antigen	Vendor or Source	Catalog #	Working concentration	Applications

S100A4	Abcam	ab197896	1:1000; 1:250	WB, IF
S100A4	CST	13018S	1:50	IP
α-SMA	Abclonal	A17910	1:1000; 1:250	WB, IF
LOX	Abcam	ab174316	1:1000; 1:250	WB, IF
Flag-Tag	MBL	M185-3	1:1000; 1:50	WB, IP
DDDDK-Tag	Abclonal	AE092	1:1000	WB
IgG	Abcam	ab131368	1:1000	WB
HA-Tag	MBL	M180-3	1:1000; 1:50	WB, IP
HA-Tag	Abclaonl	AE036	1:1000	WB
GAPDH	Proteintech	10494-1-AP	1:5000	WB
β-tubulin	Abclonal	A12289	1:1000	WB
Elastin	Bioss	Bs-1756R	1:250	IF
Myocardin	Sigma-Aldrich	SAB2106915	1:1000	WB
SM22α	Proteintech	10493-1-AP	1:1000; 1:250	WB, IF
Calponin	Abcam	ab46794	1:5000	WB
COL1	Novus	NBP1-30054	1:1000; 1:100	WB, IF
Anti-mouse IgG, HRP-linked antibody	Proteintech	SA00001-1	1:5000	WB
Anti-rabbit IgG,	Proteintech	SA00001-2	1:5000	WB

HRP-linked antibody	

Table S4. Cultured Cells

Name	Vendor or Source	Sex (F, M, or unknown)	Background Strain
Vascular smooth muscle cells	Beijing Vital River Laboratory Animal Technology Co., Ltd.	Male	C57BL/6J

Table S5. Primers for RT-PCR

Gene	Sequence (5’-3’)	
Human *GAPDH*	Forward	AATGGGCAGCCGTTAGGAAA
	Reverse	GCCCAATACGACCAAATCAGAG
Human *S100A4*	Forward	TGACAAGTTCAAGCTCAACAAGTC
	Reverse	TTCAAAGAATTCGTTACACATCATG
Mouse *β-Actin*	Forward	GGCTGTATTCCCCTCCATCG
	Reverse	CCAGTTGGTAACAATGCCATGT
Mouse *S100A4*	Forward	GTGTCCACCTTCCACAAATACTC
	Reverse	CAAAGAATTCATTGCACATCATG
Mouse *Myocd*	Forward	AGGAAGTTCCGATCAGTCTTACA
	Reverse	GGTATTAAGCCTTGGTTAGCCAG
Mouse *α-SMA*	Forward	GTCCCAGACATCAGGGAGTAA
	Reverse	TCGGATACTTCAGCGTCAGGA

Mouse *SM22α*	Forward	CCAACAAGGGTCCATCCTACG
	Reverse	ATCTGGGCGGCCTACATCA
Mouse *Calponin*	Forward	TCTGCACATTTTAACCGAGGTC
	Reverse	GCCAGCTTGTTCTTTACTTCAGC
Mouse *LOX*	Forward	ACATTACGTGAACAAATAGCGG
	Reverse	GACGTGGCAGTTTGCAGTTA

Figure S1

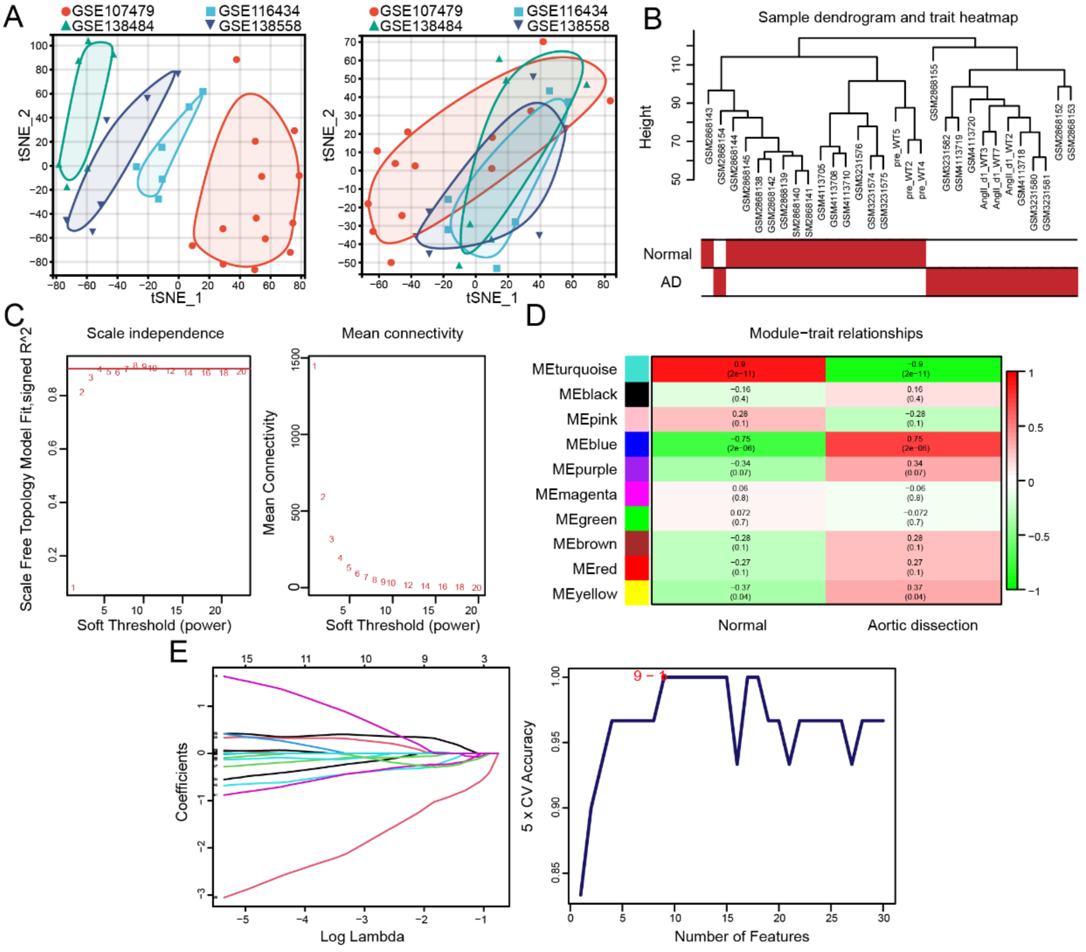
A
GSE107479
GSE116434
GSE138484
GSE138558
tSNE_1
tSNE_2
B
Sample dendrogram and trait heatmap
Height
Normal
AD
C
Scale independence
Scale Free Topology Model Fit,signed R^2
Soft Threshold (power)
Mean connectivity
Mean Connectivity
Soft Threshold (power)
D
Module−trait relationships
MEturquoise
MEblack
MEpink
MEblue
MEpurple
MEmagenta
MEgreen
MEbrown
MEred
MEyellow
Normal
Aortic dissection
E
Coefficients
Log Lambda
5 x CV Accuracy
Number of Features

Figure S2

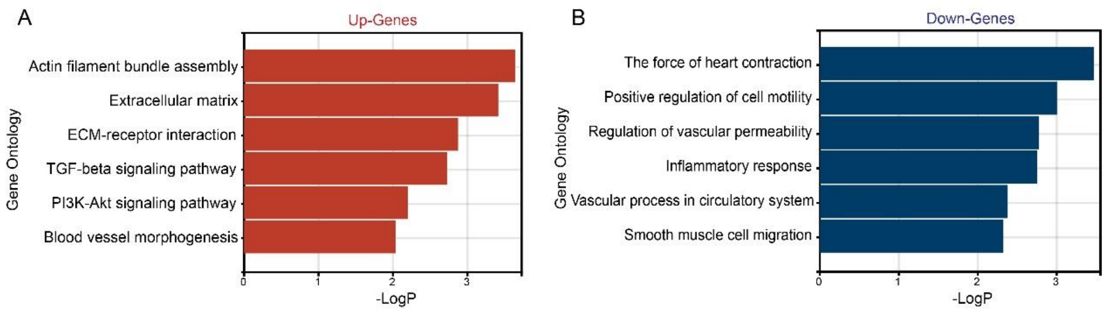
A
Up-Genes
Actin filament bundle assembly
Extracellular matrix
ECM-receptor interaction
TGF-beta signaling pathway
PI3K-Akt signaling pathway
Blood vessel morphogenesis
Gene Ontology
0
1
2
3
-LogP
B
Down-Genes
The force of heart contraction
Positive regulation of cell motility
Regulation of vascular permeability
Inflammatory response
Vascular process in circulatory system
Smooth muscle cell migration
Gene Ontology
0
1
2
3
-LogP

Figure S3

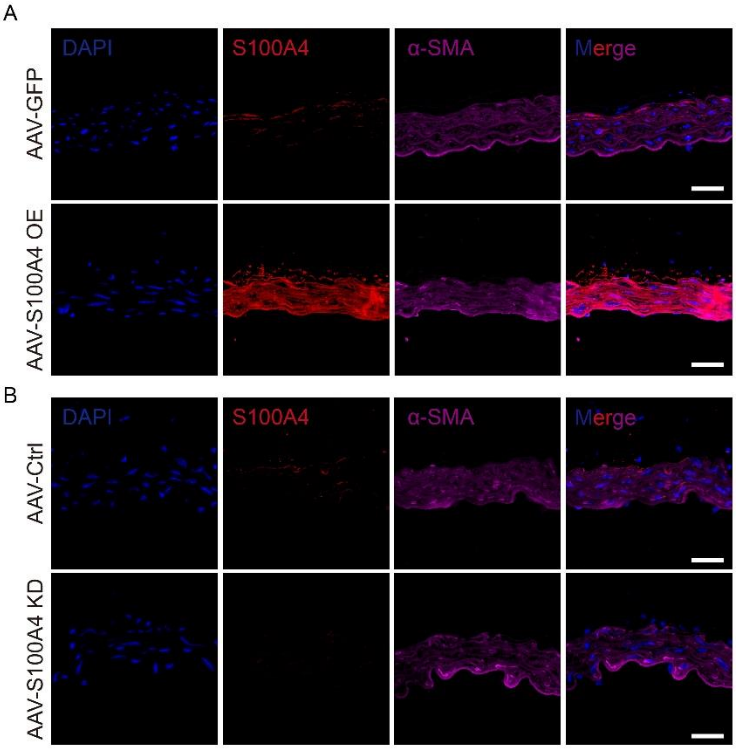
A
DAPI
S100A4
α-SMA
Merge
AAV-GFP
AAV-S100A4 OE
B
DAPI
S100A4
α-SMA
Merge
AAV-Ctrl
AAV-S100A4 KD

Figure S4

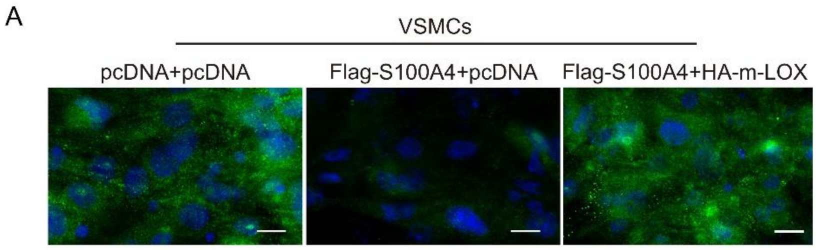

Figure S5

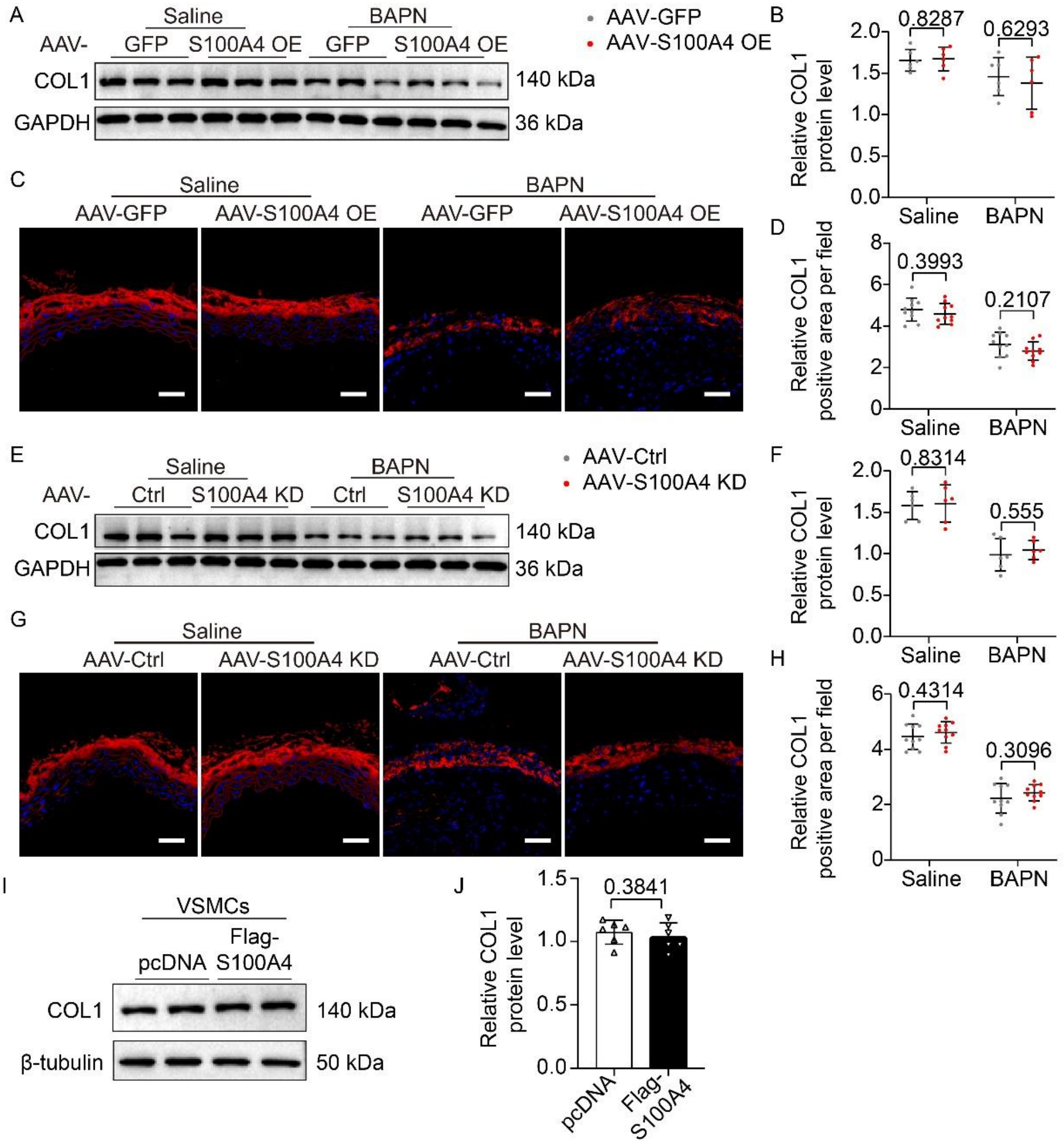

A
Saline
BAPN
AAV-
GFP
S100A4 OE
GFP
S100A4 OE
COL1
140 kDa
GAPDH
36 kDa
AAV-GFP
AAV-S100A4 OE
B
Relative COL1 protein level
0.8287
0.6293
Saline
BAPN
C
Saline
BAPN
AAV-GFP
AAV-S100A4 OE
AAV-GFP
AAV-S100A4 OE
D
Relative COL1 positive area per field
0.3993
0.2107
Saline
BAPN
E
Saline
BAPN
AAV-
Ctrl
S100A4 KD
Ctrl
S100A4 KD
COL1
140 kDa
GAPDH
36 kDa
AAV-Ctrl
AAV-S100A4 KD
F
Relative COL1 protein level
0.8314
0.555
Saline
BAPN
G
Saline
BAPN
AAV-Ctrl
AAV-S100A4 KD
AAV-Ctrl
AAV-S100A4 KD
H
Relative COL1 positive area per field
0.4314
0.3096
Saline
BAPN
I
VSMCs
pcDNA
Flag-S100A4
COL1
140 kDa
β-tubulin
50 kDa
J
Relative COL1 protein level
0.3841
pcDNA
Flag-S100A4
